# Pairwise transmembrane domain insertion during multipass protein biogenesis

**DOI:** 10.1016/j.molcel.2026.06.006

**Published:** 2026-06-24

**Authors:** Luka Smalinskaitė, Haoxi Wu, Ramanujan S. Hegde

**Affiliations:** 1 Medical Research Council Laboratory of Molecular Biology, Cambridge CB2 0QH, UK

## Abstract

The ~2,500 multipass membrane proteins encoded in the human genome are constructed mostly or entirely of transmembrane domain (TMD) pairs: exceptionally common biosynthetic units comprised of two TMDs separated by a short non-cytosolic loop. It has long been thought that each TMD of a pair sequentially enters the lipid bilayer through a lateral gate in the Sec61 protein translocation channel. Here, we show that TMD pairs can access multiple insertion routes and that most are completely impervious to small-molecule blockade of Sec61’s lateral gate. Obligate use of Sec61 is seen only for exceptional cases where the translocated loop exceeds ~60 amino acids. TMD pairs with shorter loops typically use either EMC or GEL, insertase complexes of the universally conserved Oxa1 superfamily. Our results suggest that, contrary to long-held Sec61-based models, the fundamental biosynthetic unit of nearly all multipass membrane proteins uses the Oxa1 family for insertion.

## Graphical abstract

**Graphical abstract:**
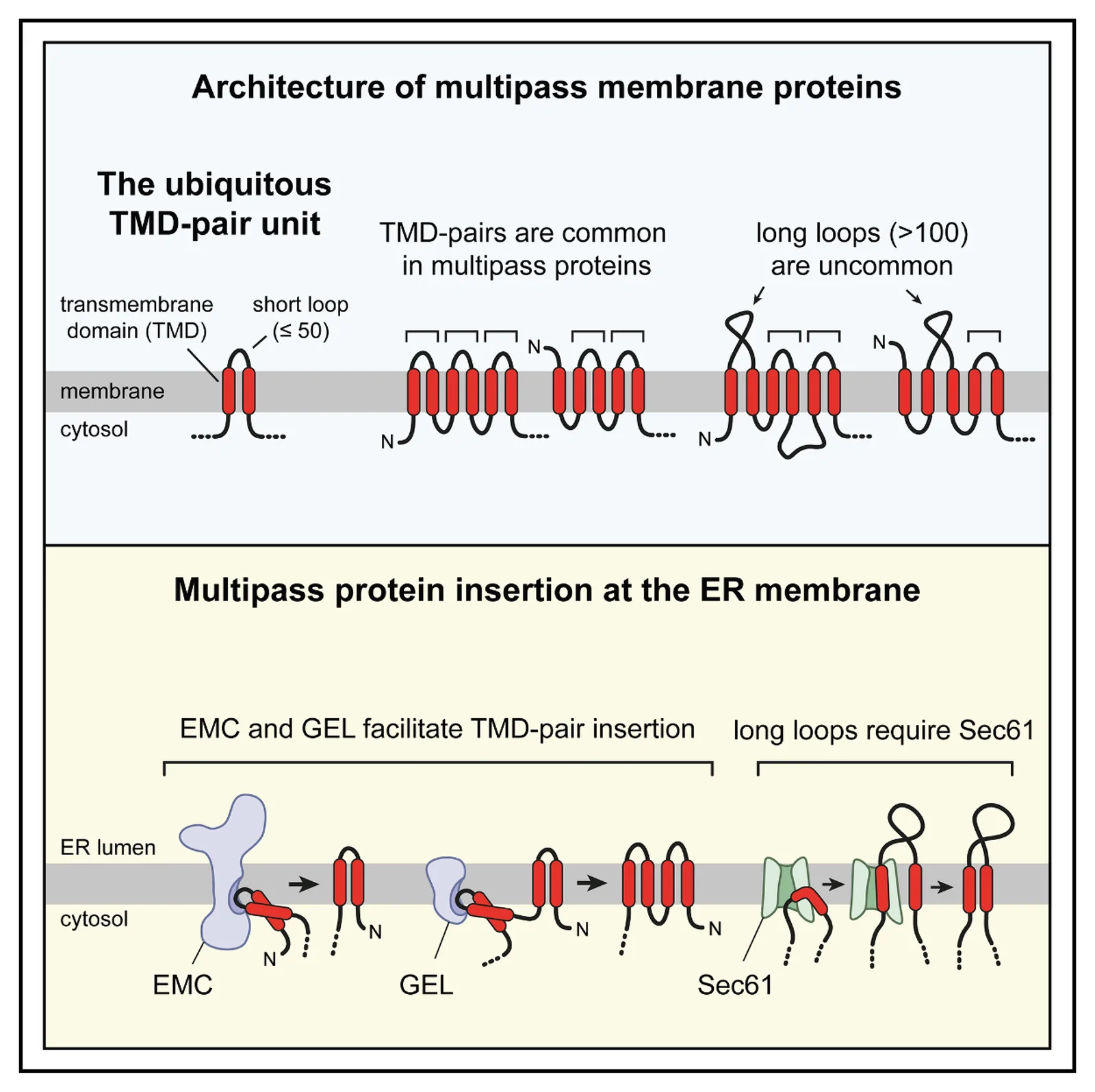

## Introduction

The human genome encodes roughly 5,000 integral membrane proteins, more than half of which are multipass membrane proteins.^1,2^ Nearly all multipass membrane proteins are inserted co-translationally into the endoplasmic reticulum (ER) membrane, where they are also modified, folded, and assembled into protein complexes to produce functional products.^3,4^ A complete mechanistic understanding of membrane protein insertion is a long-standing aim of molecular cell biology that has yet to be fully realized.

A census of multipass membrane proteins in Uniprot^5^ shows that they contain between 2 and 38 transmembrane domains (TMDs) with intervening cytosolic and non-cytosolic loops of variable length. The N terminus either faces the cytosol (termed N_cyt_) or the exoplasmic side of the membrane (termed N_exo_). Despite this wide overall diversity, the topological deconstruction of multipass proteins reveals that one biosynthetic unit occurs vastly more frequently than all others: two TMDs separated by a short (up to 50 amino acids) non-cytosolic loop.^2^ We define this biosynthetic unit as a TMD pair (sometimes called a helical hairpin^6^).

Of the ~18,000 TMDs of multipass proteins, nearly 90% of them are part of TMD pairs. For example, nearly all of the 819 G protein-coupled receptors (GPCRs) in the human genome consist of an N_exo_ TMD followed by three TMD pairs. One exception (C3AR1) contains a long extracellular inter-TMD loop of 170 amino acids, and two others each have one inter-TMD loop of 59 and 67 amino acids. Similarly, nearly all potassium and TRP (transient receptor potential) channels, ABC (ATP-binding cassette) transporters, and sodium channels are N_cyt_ proteins built entirely with TMD pairs (3, 6, and 12 TMD pairs, respectively). Thus, a major advance toward understanding multipass membrane protein biogenesis would be to delineate the insertion mechanism(s) for the ubiquitous TMD-pair biosynthetic unit.

Although postulated initially to insert spontaneously into the membrane,^6^ the current long-standing model for TMD-pair insertion involves sequential entry into the lipid bilayer via the Sec61 protein translocation channel at the ER.^7,8^ In this view, the first TMD, having just emerged from the ribosome, inserts in a “looped” configuration into a lateral gate in Sec61. In the looped state, the TMD’s N-terminal flank faces the cytosol, the C-terminal flank faces the ER lumen, and the downstream polypeptide passes through Sec61’s channel and into the ribosome exit tunnel. With continued elongation, the first TMD partitions passively into the hydrophobic lipid bilayer, the downstream loop is trans-located through Sec61 across the membrane, and the second TMD enters the channel. This second TMD then also partitions passively into the surrounding lipid bilayer via Sec61’s lateral gate. With both TMDs inserted, the ribosome-Sec61 complex returns to its starting state, ready for the next TMD pair.

The sequential lateral gate insertion model has several lines of support, although nearly all of them come from analyzing model N_cyt_ TMDs or signal peptides, whose N terminus also faces the cytosol. First, a TMD or signal peptide engaged with Sec61 in the looped configuration has been observed in several structures and by crosslinking.^9–14^ Second, TMD partitioning from Sec61 into lipid is hydrophobicity-dependent.^15–18^ Third, Sec61 inhibitors that bind and block the lateral gate^19,20^ prevent insertion of signal peptides and N_cyt_ TMDs.^21–26^ Fourth, purified Sec61 in synthetic liposomes or in detergent can engage and insert model signal peptides and N_cyt_ TMDs.^27,28^ From these findings, it is generally assumed that the subsequent steps of loop translocation and insertion of a downstream TMD would proceed via Sec61’s channel and lateral gate.

Although compelling, the sequential lateral gate insertion model is difficult to rationalize for most native multipass proteins whose TMDs are often relatively hydrophilic. The calculated free energy of membrane partitioning (ΔG_app_, where negative values indicate favored insertion^17^) is disfavored for most multipass TMDs.^2^ Hence, many TMDs from multipass proteins cannot effectively engage Sec61 or pass into the membrane when tested in isolation *in vitro* but are inserted when produced in their native context with adjacent TMDs.^18,29–32^ Recent experiments with broad-acting Sec61 inhibitors show that several topologically diverse multipass membrane proteins from different families are refractory to inhibition in cells and *in vitro* despite containing TMD pairs.^33,34^ Finally, insertion occurs co-translationally, so delays in Sec61 engagement, as might occur for sub-optimal TMDs, would result in the emergence of the second TMD of a TMD pair before the first TMD had been recognized. This can result in competition and aberrant or failed insertion.^35,36^

To understand how TMD pairs are inserted, we have investigated a set of diverse native pairs whose TMDs vary widely in their properties. Unlike the long-standing model of sequential insertion via the Sec61 lateral gate, we find that most TMD pairs can instead insert efficiently by lateral gate independent routes, often involving the Oxa1 family insertases EMC and GEL. These results are consistent with a recently proposed unifying model for membrane protein biogenesis^37^ and have major implications for the molecular basis of multipass membrane protein insertion.

## Results

### TMD pairs can be inserted by a Sec61-independent mechanism

**Figure 1:**
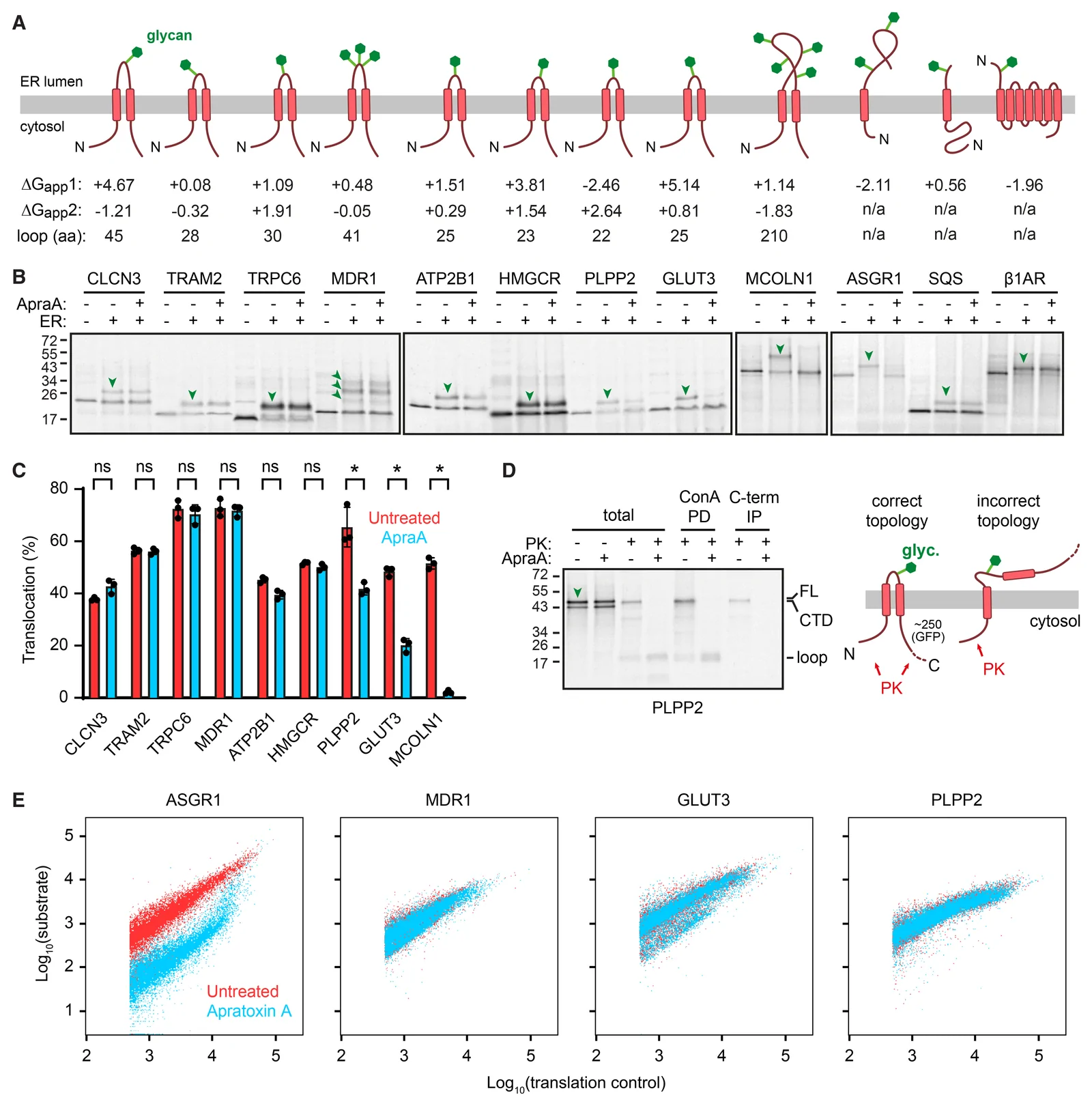
TMD-pair insertion is refractory to Sec61 inhibition (A) Diagrams of key membrane protein constructs used in this study. The predicted free energy for membrane insertion (ΔG_app_ in kcal/mol) of the first one or two TMDs is indicated, as is the length of the translocated loop for TMD pairs. (B) The constructs in (A) were translated in rabbit reticulocyte lysate (RRL) containing ^35^S-methionine in the absence or presence of ER-enriched membranes (ER) from Expi293F cells. The Sec61 lateral gate inhibitor Apratoxin A (ApraA) was included where indicated. Translation products were analyzed by SDS-PAGE and autoradiography. Glycosylated products are marked by green arrowheads. (C) Quantification of translocation (mean ± SD) from three independent experiments as in (B). *p < 0.001 by one-way ANOVA, followed by Tukey’s test. (D) The PLPP2 TMD pair, followed by GFP, was translated in RRL containing ^35^S-methionine and ER-enriched membranes in the absence or presence of ApraA. Membranes were isolated and treated with proteinase K (PK), where indicated, and analyzed directly (total) or after concanavalin A pull-down (ConA PD) or GFP immunoprecipitation (C-term IP). Full-length product (FL), the protease-protected C-terminal domain (CTD), and the protease-protected TMD-pair fragment (loop) are indicated. A cartoon of the two different topological populations of PLPP2 is shown on the right. (E) Wild-type (WT) Flp-In T-REx 293 cells were transfected with inducible dual-color reporter plasmids to monitor the stability of the indicated proteins. Reporter plasmids encode indicated proteins fused to a fluorescent protein and a tandem translation control of a second fluorescent protein, separated by a ribosome-skipping P2A sequence. 16–24 h after transfection, expression was induced with doxycycline in the presence or absence of ApraA. 6 h after induction, cellular fluorescence was monitored by flow cytometry. See also Figures S1 and S2.

To investigate the Sec61 lateral gate insertion model for TMD pairs, we utilized Apratoxin A (ApraA), a potent Sec61 inhibitor that binds to and obstructs the lateral gate.^20,26^ We chose a panel of eight TMD-pair substrates derived from the first two TMDs of a diverse set of N_cyt_ multipass membrane proteins. Each substrate contained two TMDs, their flanking cytosolic domains (~10 amino acids each), the translocated loop, an epitope tag at the N terminus, and a 50-amino-acid-long segment of green fluorescent protein (GFP) at the C terminus (Figure 1A; Figure S1). The sequences, hydrophobicity of the TMDs, and length of the translocated loop are indicated. To monitor correct translocation, a glycosylation site was introduced into the loop of three substrates that did not contain any: ATP2B1, HMGCR, and PLPP2.

*In vitro* translation in reticulocyte lysate in the presence of mammalian ER membranes resulted in each of the TMD-pair constructs being glycosylated (downward green arrows; Figures 1B and 1C), seen as a membrane-dependent ~4 kD shift per glycan. Surprisingly, six of eight substrates were completely refractory to Sec61 inhibition by ApraA, with the remaining two (PLPP2 and GLUT3) being inhibited by ~50%. By contrast, a substrate derived from the first two TMDs of Mucolipin1 (MCOLN1), which contains a ~200 amino acid long translocated loop, was completely inhibited by ApraA, as seen before.^26^ As expected from earlier studies,^33^ insertion of a model N_cyt_ TMD from the asialoglycoprotein receptor (ASGR1) was potently inhibited by ApraA, which had no effect on the insertion of a tail-anchored protein (squalene synthase, or SQS) and an N_exo_ signal anchor (from the GPCR β1AR).

Further investigation of the partial Sec61 dependence for PLPP2 and GLUT3 revealed that in both cases, the inhibited population was in an incorrect single-pass N_cyt_ topology. This conclusion emerged from protease-protection experiments using variant constructs with a longer C-terminal tail to facilitate analysis. This experiment showed that these two proteins adopt two distinct topologies (Figure 1D; Figure S2A). The correct topology spans the membrane twice, has both the N and C terminus facing the cytosol, and produces a small protease-protected fragment (labeled “loop” in Figure 1D) that is glycosylated (i.e., binds to the lectin concanavalin A) but not recovered by immuno-precipitation (IP) via the C-terminal domain. The incorrect topology spans the membrane once, exposes only the N terminus to the cytosol, and produces a near-full-length protease-protected product (labeled “CTD” [C-terminal domain] in Figure 1D) that was glycosylated and recovered by C-terminal IP. With ApraA, the incorrect single-pass topology was completely lost (i.e., no CTD fragment), whereas the correct double-pass topology (i.e., the loop fragment) was either unchanged (GLUT3) or increased (PLPP2). As expected, TRAM2 was made only in the correct double-pass topology, which was fully resistant to ApraA. These results indicate that to the extent PLPP2 and GLUT3 engage the Sec61-dependent route for insertion, the resulting product is quantitatively incorrect, with the correct product being generated by a mechanism refractory to ApraA inhibition.

Consistent with the *in vitro* results, biogenesis in cultured cells of fluorescent-protein-tagged TRAM2, ANO6, MDR1, GLUT3, and PLPP2 was resistant to ApraA, whereas ASGR1 was strongly impaired by ApraA (Figure 1E; Figure S2B; Sundaram et al.^34^). In this assay,^38^ the protein of interest is fused to a fluorescent protein, with a second fluorescent protein separated by a ribosome-skipping P2A sequence serving as an expression control. Hence, failed biogenesis results in degradation of the first fluorescent protein relative to the expression control. As none of the multipass reporter proteins were affected by ApraA, we conclude that their complete biogenesis, including the topologically correct insertion of the first TMD pair of these proteins, can proceed efficiently despite a blocked Sec61 lateral gate.

### TMD-pair insertion operates differently than their individual TMDs

**Figure 2:**
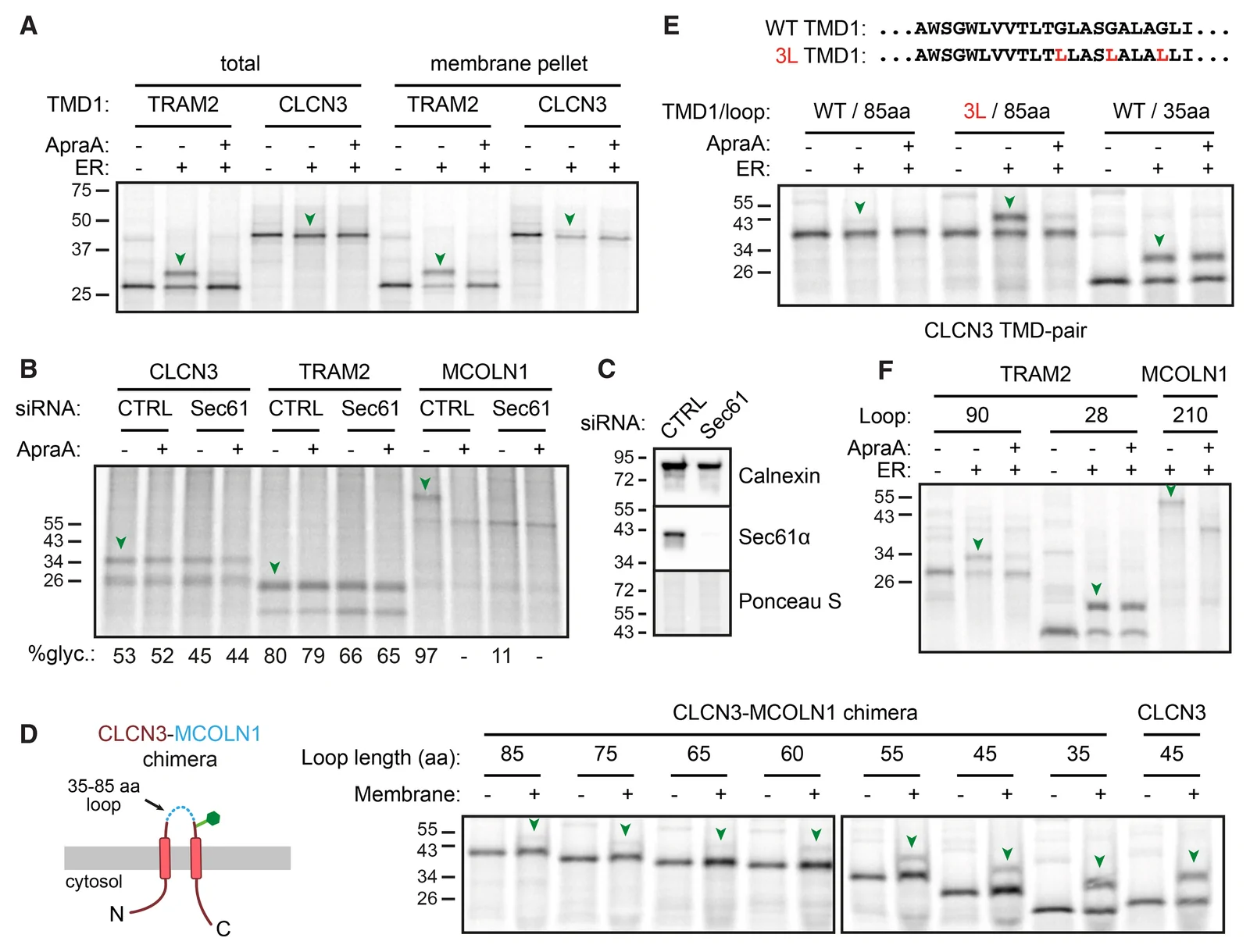
The TMD pair is a distinct biosynthetic unit (A) Reporter constructs containing only the first TMD of either TRAM2 or CLCN3 were analyzed as in Figure 1B (total) and after isolating ER membranes (membrane pellet). Green arrowheads indicate glycosylated products. (B) Semi-permeabilized cells (SPCs) produced from Flp-In T-REx 293 cells treated with the indicated small interfering RNAs (siRNAs) for 72 h were used immediately for *in vitro* translocation reactions of CLCN3, TRAM2, or MCOLN1 constructs (Figure 1A) as in Figure 1B. Pelleted SPCs were analyzed. Percent glycosylation was quantified for each lane. (C) Immunoblot of SPCs from (B), with Ponceau S-stained membrane also shown. (D) Left: cartoon of CLCN3-MCOLN1 chimeric reporters with various loop lengths. Right: CLCN3-MCOLN1 chimeras or the native CLCN3 TMD pair were translated in RRL containing ^35^S-methionine in the absence or presence of WT SPCs, and the total translation products or pelleted membranes were analyzed by SDS-PAGE. (E) Top: CLCN3’s TMD1 sequence and the 3L mutant. Bottom: CLCN3 TMD-pair reporters with the indicated TMD1 and loop lengths were translated and analyzed as in Figure 1B. (F) Reporters containing the first two TMDs of TRAM2 (with two different loop lengths) and MCOLN1 were translated and analyzed as in Figure 1B. See also Figure S3.

Given the unexpected result for TMD pairs relative to the model N_cyt_ TMD from ASGR1, we dissected the insertion reaction more finely by analyzing constructs containing only the first TMD of two different TMD pairs, TRAM2 and CLCN3. In the case of TRAM2, whose first TMD is a canonical hydrophobic helix with a predicted ΔG for insertion of +0.08 (Figure 1A; Figure S1B), insertion into the ER *in vitro* was efficient and potently inhibited by ApraA (Figure 2A). By contrast, the first TMD of CLCN3, which has substantial hydrophilic character and a predicted ΔG of - +4.67, produced a barely detectable inserted population. The small population of inserted CLCN3 TMD1 was nonetheless inhibited by ApraA.

The differential sensitivity to ApraA of TMD1 alone versus the TMD pair indicates that TMD-pair insertion is not simply a sequential process in which TMD1 inserts via the Sec61 lateral gate, followed by loop translocation and TMD2 insertion. Instead, insertion of the pair can proceed by an ApraA-insensitive mechanism that does not depend on TMD1 engaging and passing through the Sec61 lateral gate. Furthermore, the imperviousness of TMD-pair insertion to ApraA for TRAM2 and CLCN3 is not due to TMD1 being resistant to Sec61 inhibition but rather the TMD pairs evidently following a Sec61-independent route.

This conclusion was confirmed and extended by translocation assays into ER membranes from cells following acute Sec61 knockdown (KD). As expected, the ApraA-sensitive substrate MCOLN1 failed to be translocated into the Sec61-KD ER (Figures 2B and 2C). By contrast, TMD pairs from CLCN3 and TRAM2 were unaffected by Sec61-KD, even when further supplemented with ApraA. Thus, TMD pairs can be efficiently inserted via a Sec61-independent route even though TMD1 isolated from such pairs is inserted by a strictly Sec61-dependent route (albeit inefficiently in the case of CLCN3).

These experiments also revealed that TMD2 can markedly improve the insertion of an otherwise inefficiently inserting TMD1, such as that from CLCN3 (compare Figure 2A with Figure 1B). The apparent cooperativity of two TMDs during Sec61-independent insertion is noteworthy because the first TMD of many N_cyt_ multipass membrane proteins is of moderate hydrophobicity with predicted ΔG values well above 0, similar to CLCN3. It has long been unclear how TMDs that cannot effectively insert in isolation via Sec61^17,18^ can nonetheless insert efficiently in their native context. The example of CLCN3 suggests that one answer is the use of a Sec61-independent route that permits cooperation with an adjacent TMD.

### Sec61 is strictly required for the translocation of long inter-TMD loops

A key topological feature of TMD pairs is a relatively short intervening translocated loop. By contrast, N_cyt_ single-pass proteins typically have long translocated domains and are strictly Sec61-dependent (the exceptions being tail-anchored proteins, which have short translocated domains and are inserted by Sec61-independent mechanisms^39–41^). Similarly, two TMDs separated by a long translocated loop (such as MCOLN1) would initially behave like an N_cyt_ single-pass protein because a long period of time would elapse before the second TMD emerges from the ribosome. We therefore posited that the key determinant for Sec61-independent insertion of a TMD pair is its short loop.

Focusing on CLCN3, we found that varying the loop length (using sequence from the loop of MCOLN1) resulted in a progressive loss of insertion beyond ~60 amino acids, with only a small amount of insertion seen with an 85 amino acid loop (Figure 2D). To the extent that insertion was observed with the 85-residue loop substrate, it was inhibited by ApraA, in contrast to the protein with a 35-residue loop (Figure 2E). Insertion of the 85-residue loop substrate could be markedly improved by increasing the hydrophobicity of TMD1 with three glycine-to-leucine substitutions. This too was potently inhibited by ApraA.

Similar results were seen with TRAM2, where replacement of its native 28-residue loop with a 90-residue loop converted the construct from an ApraA-insensitive protein to an ApraA-sensitive protein (Figure 2F). In this case, no adjustments were needed to TMD1 to maintain high insertion efficiency with a long loop because it has sufficient hydrophobic character (ΔG of +0.08). These results indicate that insertion of two TMDs separated by a loop longer than ~60 amino acids obligately proceeds via the Sec61 lateral gate, which is initially engaged and opened by TMD1 to initiate loop translocation.

Because the constructs insensitive and sensitive to ApraA have the same TMDs, their differential sensitivities to Sec61 blockade cannot be ascribed to inherent ApraA resistance of these TMDs. Instead, it is the short intervening loop that allows TMD pairs to insert via a Sec61-independent mechanism, whereas a long loop imposes strict Sec61 dependence. Notably, the Sec61-independent route seems to tolerate significantly lower hydrophobicity in the two TMDs than Sec61-mediated insertion. Hence, TMD1 from CLCN3 and TMD2 from PLPP2 (and GLUT3) are insertion competent in their TMD-pair contexts via the Sec61-independent route but fail insertion by the Sec61-dependent route. Consistent with this idea, multipass TMDs adjacent to long translocated domains have a median negative ΔG for insertion (i.e., favored insertion), whereas TMDs adjacent to short translocated loops have a median ΔG around +1 (i.e., strongly disfavored for Sec61-mediated insertion) (Figure S3).

### TMD pairs can be translocated as a unit

The fact that insertion of a TMD pair is mechanistically distinct from the first TMD alone strongly suggests that the two TMDs do not insert sequentially. The further observation that TMD2 of a pair (in the case of CLCN3) can markedly improve insertion of TMD1 raised the possibility that a TMD pair may insert as a unit, a model that is plausible because TMD2 would emerge from the ribosome within ~10 s after TMD1 (assuming a translation rate of ~5–6 amino acids per second). We tested this idea in two ways: (1) by asking whether the intervening loop between the two TMDs fully emerges from the ribosome prior to initiation of translocation and (2) whether a fully emerged TMD pair is capable of insertion.

**Figure 3:**
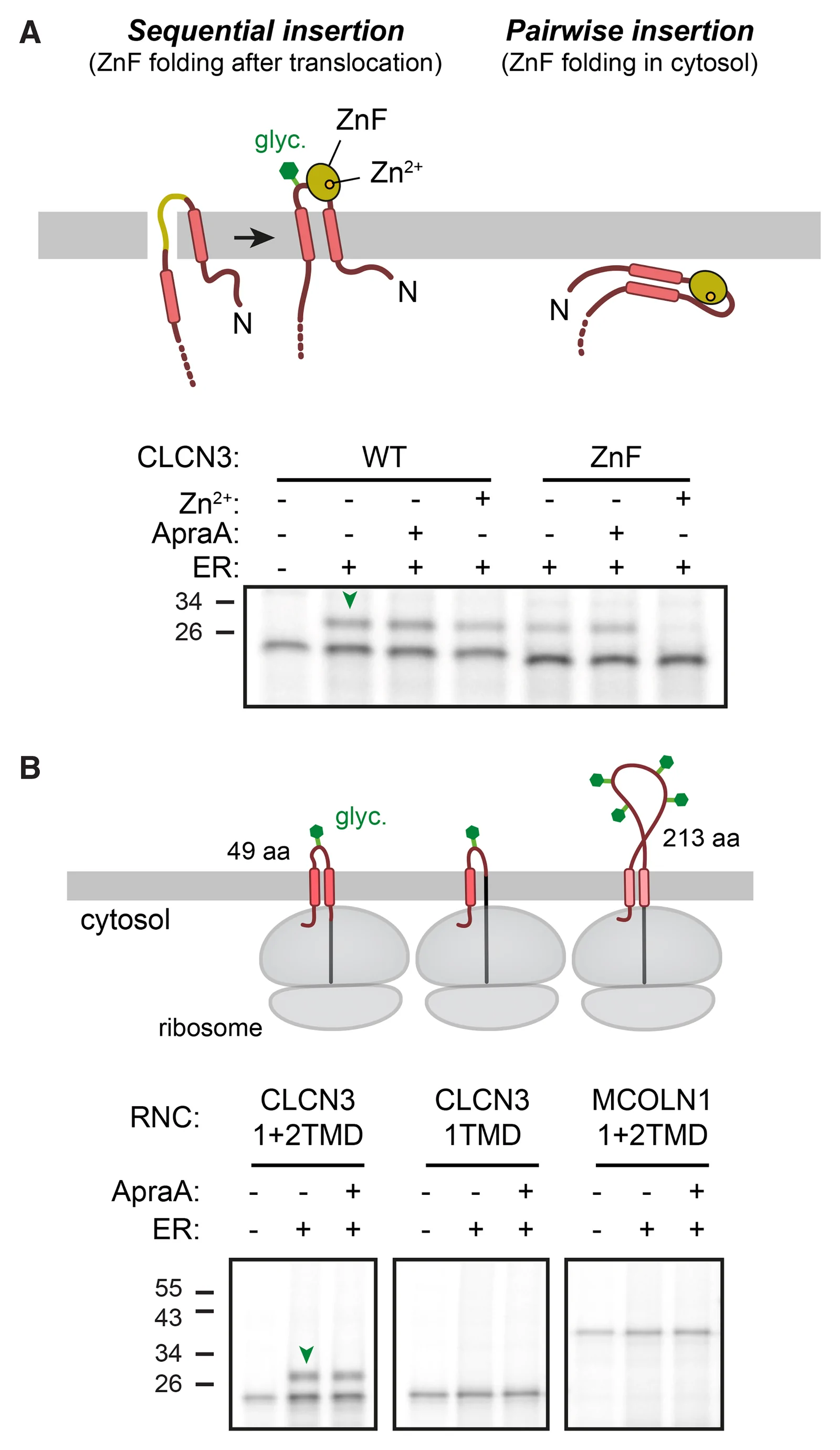
The TMD-pair inserts as a unit (A) Top: cartoon of predictions from the sequential and pairwise insertion models for a TMD-pair containing a zinc finger domain (ZnF, yellow) in the translocated loop. Bottom: CLCN3 TMD pairs without (WT) or with ZnF were translated in RRL containing ^35^S-methionine in the absence or presence of WT SPCs. Zn^2+^ or ApraA was included in the reaction where indicated. Samples without SPCs were analyzed directly, and samples containing SPCs were analyzed after pelleting the membranes. (B) Top: cartoon of ribosome-nascent chain complexes (RNCs) analyzed. Bottom: each construct was translated in RRL containing ^35^S-methionine, then incubated without or with SPCs. Samples without SPCs were analyzed directly, and samples containing SPCs were analyzed after pelleting the membranes.

In the first experiment, we replaced a segment of the loop in CLCN3 with a zinc finger (ZnF), a 29-residue domain that folds into a compact, non-translocatable structure when Zn^2+^ is present.^42^ It has previously been shown that folding of the ZnF depends on the entire domain having emerged from the ribosome exit tunnel.^43^ Thus, if translocation has been initiated before this point, the ZnF can pass through the membrane and fold on the trans side (Figure 3A, left). By contrast, emergence and folding of the ZnF in the cytosol would impede translocation (Figure 3A, right). We found that the insertion of ZnF-containing CLCN3 was potently inhibited by Zn^2+^, which had no effect on the translocation efficiency of native CLCN3 (Figure 3A). Zn^2+^ modestly inhibited translation in both cases, and ZnF partially reduced translocation efficiency even without added Zn^2+^, probably due to some endogenous Zn^2+^ in the translation system. These slight artifacts notwithstanding, the potent inhibition of translocation in a ZnF and Zn^2+^-dependent manner suggests that the loop fully emerges from the ribosome prior to translocation. Thus, TMD1 does not initiate translocation immediately after its emergence from the ribosome, as was proposed based on assays using a series of stalled ribosome-nascent chain complexes (RNCs) of various lengths.^16,44,45^

In the second experiment, we prepared a stalled RNC in which the full TMD pair of CLCN3 had emerged from the ribosome, then incubated this isolated complex with ER membranes and monitored insertion by glycosylation. For comparison, a length-matched construct in which TMD2 was replaced by a soluble segment of polypeptide was analyzed. The RNCs in this staged insertion reaction behaved very similarly to non-truncated constructs translated in the presence of ER: the TMD pair inserted far more efficiently than the TMD1-alone construct, with only the pair being refractory to inhibition by ApraA (Figure 3B). Because the RNC insertion reaction was not coupled to ongoing translation, this experiment gave TMD1 ample time to engage translocation machinery. The fact that insertion of TMD1 was nonetheless inefficient in the absence of TMD2 indicates that TMD2 directly aids TMD1 insertion during the concerted insertion of this TMD pair, which proceeds via a Sec61-independent mechanism. Two closely spaced TMDs in a potassium channel have been shown to interact in the ribosome exit tunnel,^46^ supporting the concept of TMD-pair insertion as a single unit.

### EMC directly facilitates TMD-pair insertion

The major known alternatives to Sec61-mediated insertion are the Oxa1 superfamily of insertases. Furthermore, the length of the translocated loop for TMD pairs is similar to the length limit of previously characterized Oxa1-family insertion reactions of N-tails and C-tails.^47,48^ The mammalian ER membrane contains three Oxa1 family insertase complexes known as GET, EMC, and GEL.^33,39,49,50^ Of these, access to GET requires a highly specific and dedicated targeting factor (known as GET3 or TRC40; Stefanovic and Hegde^41^), which only engages tail-anchored proteins with TMDs of high hydrophobicity.^39,51^ Given its broad conservation across all eukaryotes, we first focused on EMC, using CLCN3 as a model TMD pair.

**Figure 4:**
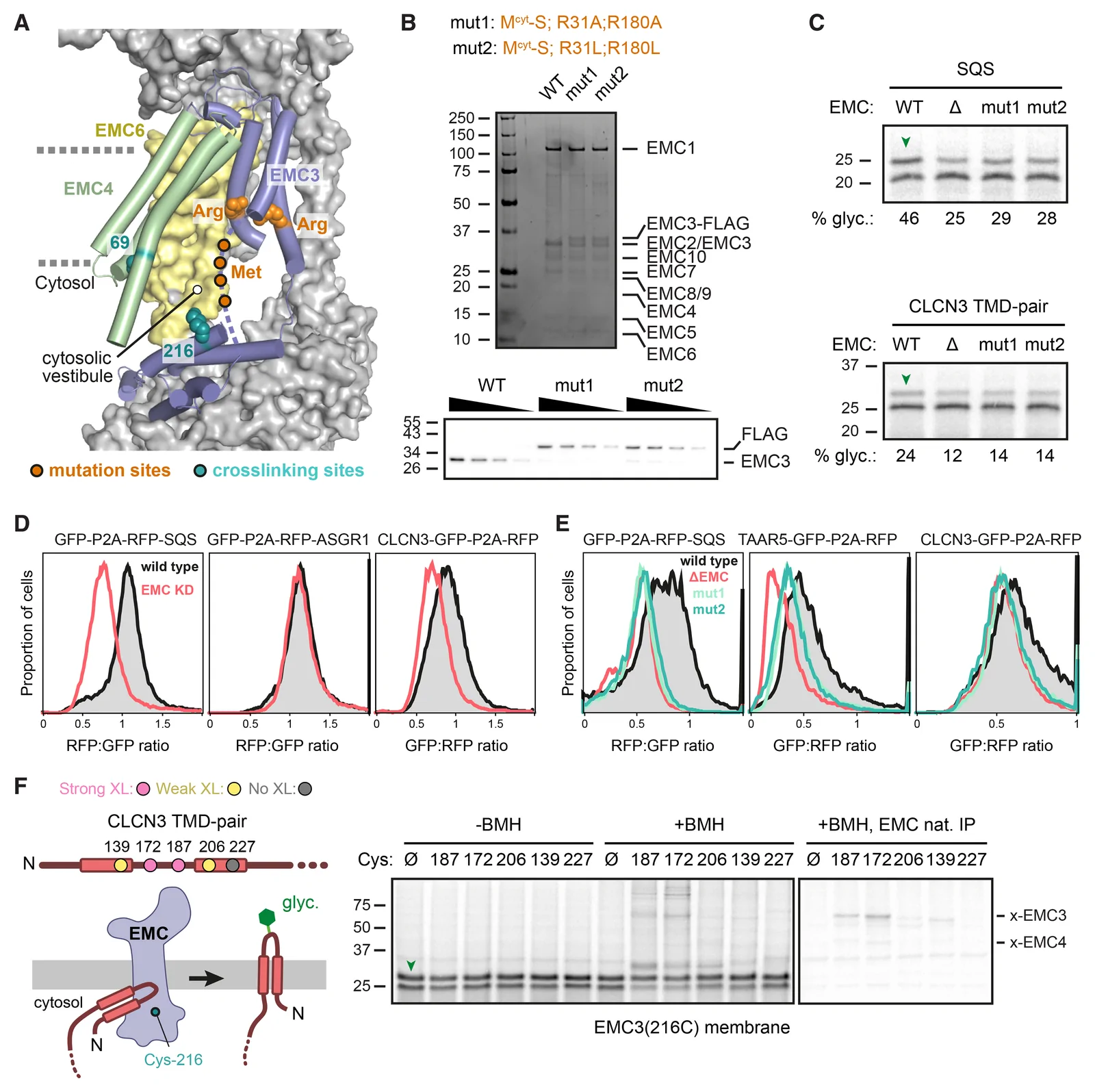
EMC can directly insert TMD pairs (A) Positions in EMC that were crosslinked to substrates (teal), and positions that were mutated to impair EMC-mediated insertion (orange) mapped onto an EMC structure (PDB: 7ADO). The methionine residues are in a flexible cytosolic loop of EMC3 (dashed line) that is not resolved in the structure. (B) Top: purifications of WT and two mutant (mut) versions of EMC3 under native conditions show equal expression levels and co-purification of all other EMC subunits. Bottom: blot of EMC3 in WT or cells after long-term overexpression of EMC mutants shows comparable expression and effective replacement of endogenous EMC3 to ~90%. Two-fold serial dilutions were analyzed for each sample. (C) ^35^S-methionine-labeled SQS or CLCN3 TMD-pair RNCs were added to ER membranes prepared from the indicated cells. The pelleted ER membranes were analyzed. Green arrowheads indicate glycosylated products. (D) Cells stably integrated with the indicated inducible dual-color fluorescent reporters were treated with control or EMC3 siRNA for 72 h. After reporter induction with doxycycline for 6 h, fluorescence was analyzed using flow cytometry. The ratio of substrate fluorescence to fluorescence of the translation control was plotted as a histogram for at least 10,000 cells. (E) WT, ΔEMC, or EMC mutant cell lines were transiently transfected with the indicated inducible dual-color fluorescent reporters and analyzed as in (D). (F) Left: cartoon of site-specific crosslinking strategy between single cysteine-containing CLCN3 TMD-pair constructs and EMC containing Cys-216 in EMC3. Right: ^35^S-methionine-labeled CLCN3 TMD-pair variants were translated in the presence of EMC3(216C) ER membrane. Isolated ER membrane was untreated (-BMH) or crosslinked with BMH (+BMH). The left gel shows total reaction products, and the right gel shows non-denaturing EMC immunoprecipitation (nat. IP). Crosslinks to EMC3 and to EMC4 are indicated. The higher molecular weight crosslinks from positions 172 and 187 in the translocated loop are to ER lumenal chaperones. See also Figure S4.

Two conserved regions in the Oxa1 family member EMC3, a cytosolic methionine loop and membrane-embedded hydrophilic vestibule (Figure 4A), are thought to facilitate insertion of N- and C-tails.^48,52–54^ To test their importance for TMD-pair insertion, we made two mutants in which methionine residues in the cytosolic loop were changed to serine residues, and arginine residues in the hydrophilic vestibule were changed to either alanine or leucine. Both mutants assembled correctly as judged by their efficient replacement of endogenous EMC3 during long-term overexpression, and association with all other EMC sub-units similar to wild-type EMC (Figure 4B). When the CLCN3 TMD pair was translated in the presence of mutated ER membranes, both mutants showed an insertion defect similar to EMC6-knockout (KO) ER membranes (which lack most EMC subunits due to their degradation^39,55^). CLCN3 impairment was comparable to the EMC substrate SQS (Figure 4C). Thus, the same regions of EMC that facilitate SQS insertion are important for TMD-pair insertion.

In cells, we found that full-length CLCN3 was partially but significantly destabilized when expressed in EMC-KD cells (lacking the core EMC subunit EMC3) (Figure 4D). As expected, SQS and β1AR were both affected by EMC KD, whereas ASGR1 was not (Figures 4D, S4A, and S4B). Furthermore, CLCN3 was destabilized to similar levels in EMC-KO cells and cells stably expressing either of the EMC mutants when monitored with fluorescent reporters (Figure 4E). As expected, these mutant-expressing cells impaired the biogenesis of previously established EMC insertase substrates SQS and TAAR5. These findings in cells corroborate the *in vitro* experiments, suggesting that the same regions of EMC involved in N- and C-tail insertion also mediate TMD-pair insertion.

During N- and C-tail insertion, the translocated tail is temporarily housed in the cytosol-facing vestibule prior to translocation across the membrane.^48,56^ To determine whether the translocated loop of a TMD pair follows a similar route, we monitored crosslinking to EMC3 containing a single unique cysteine introduced at position 216, located at the entry to the hydrophilic vestibule (see Figure 4A). This EMC variant is fully functional and crosslinks to pre-translocated N- and C-tails of known EMC substrates.^48,56^ When single cysteine-containing variants of the CLCN3 TMD pair were translated *in vitro* in the presence of EMC(C216) ER membranes, a sulfhydryl crosslinking agent (bismaleimidohexane, BMH) captured crosslinks between CLCN3 and EMC3 (Figure 4F). The crosslinks to EMC3 were most effective from the inter-TMD loop, with weak crosslinks visible from the loop-proximal positions of TMD1 and TMD2 and no cross-linking from a distal site in TMD2.

A similar pattern of crosslinks, albeit much weaker, to an endogenous cytosol-facing cysteine in EMC4 (see Figure 4A) was also seen (Figure 4F). These crosslinks between CLCN3 and EMC occurred only from the non-glycosylated population of CLCN3 (Figure S4C), suggesting that the observed interaction occurs prior to loop translocation, consistent with the cytosol-facing crosslinking sites on EMC3 and EMC4. Thus, CLCN3’s TMD pair depends on EMC’s insertase activity for insertion *in vitro*, CLCN3 depends on EMC for efficient biogenesis in cells, and the TMD pair is in physical proximity to EMC3’s insertion path prior to insertion. We conclude from these data that EMC can directly insert the CLCN3 TMD pair by facilitating translocation of the inter-TMD loop via EMC’s hydrophilic vestibule.

### GEL and EMC are partially redundant insertases for TMD pairs

**Figure 5:**
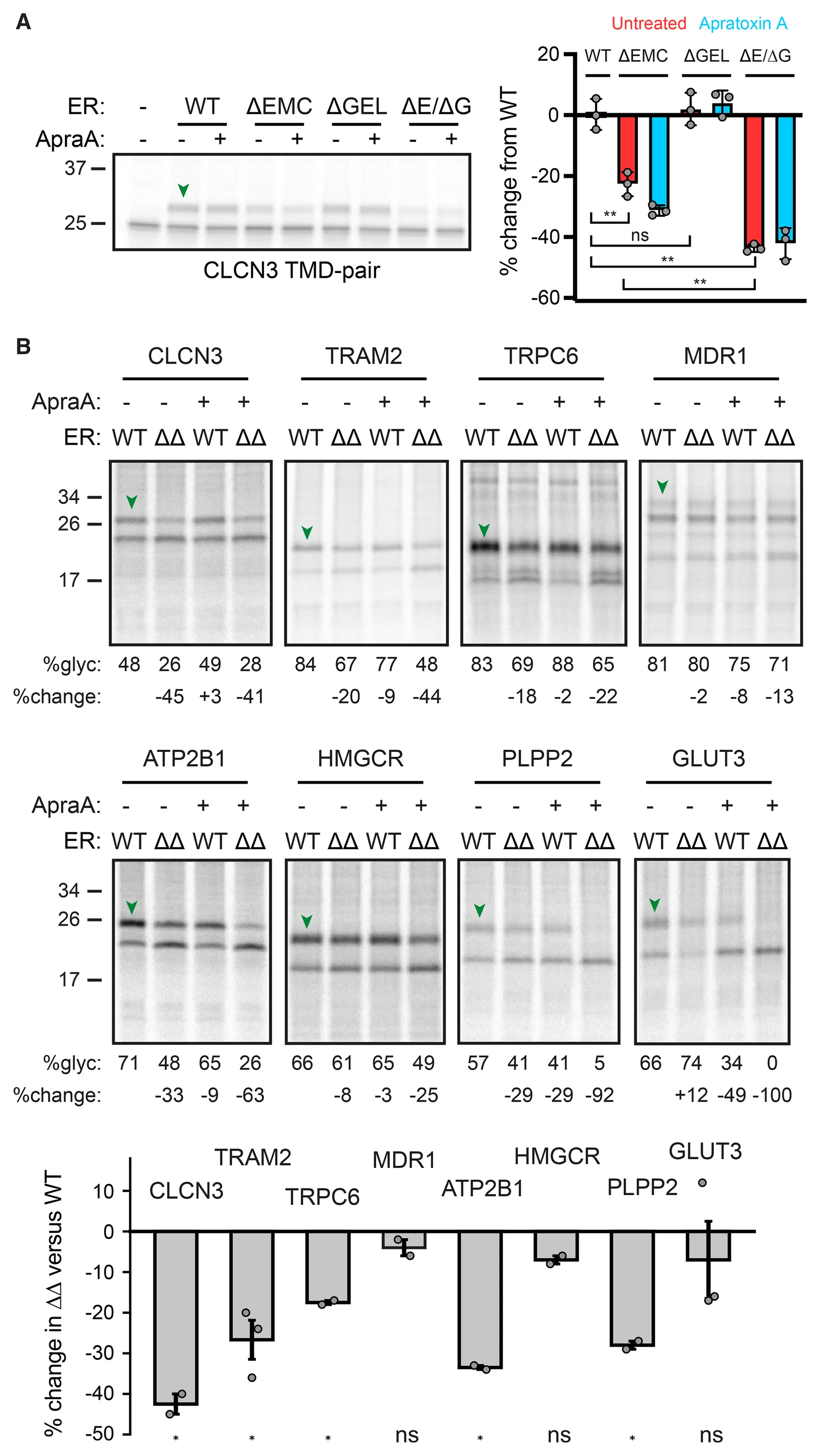
GEL and EMC redundantly facilitate TMD-pair insertion (A) Left: CLCN3 TMD pair was analyzed as in Figure 1B using SPCs as the source of ER membranes. The SPCs were from WT, ΔEMC, ΔGEL, or ΔEMC/ΔGEL dKO cells (ΔE/ΔG). Glycosylated products are indicated by green arrowheads. Right: quantification of percent insertion change compared with WT (mean ± SD; *n* = 3). Double asterisks indicate *p* < 0.01 using two-tailed Welch’s *t* tests; ns indicates not significant. (B) TMD pairs were analyzed as in (A) using WT or EMC/GEL dKO (ΔΔ) cells. Percent glycosylation and percent change in glycosylation from WT are indicated below each lane. The graph shows the percent change in ΔΔ compared with WT (mean ± SD; *n* = 3). Asterisk indicates *p* < 0.05 using two-tailed Welch’s *t* tests; ns indicates not significant.

CLCN3 TMD-pair insertion, as monitored by glycosylation, was further impaired into ER from double-KO (dKO) cells lacking both EMC and GEL (knocked out for the core subunit TMCO1, which leads to degradation of its partner OPTI^49^) (Figure 5A). Similar results were seen for the CLCN3 TMD pair lacking a glycosylation site, with insertion being monitored by a protease-protection assay (Figure S5A). Although insertion into dKO ER showed a strong impairment of CLCN3 insertion, it was not complete. Notably, however, the residual insertion seen with dKO ER was still not inhibited by ApraA, indicating that even when the major Oxa1 family routes of insertion are not available, Sec61 still does not become an obligate insertion route. This is consistent with the finding that TMD1 is a very poor Sec61 substrate. The basis of residual insertion in dKO cells remains to be elucidated but could be an unassisted mechanism, a yet-un-identified insertase, or the GET complex being used in an unconventional manner. GLUT3 and TRAM2 also showed a greater insertion deficiency into the ER of dKO cells than single-KO cells, supporting the idea that EMC and GEL have partially redundant roles (Figure S5B).

Using the dKO cells, we tested the other TMD-pair constructs and found they were variably dependent on Oxa1 insertases (Figures 5B and S5C). Partial dependence on EMC and GEL could be observed for TRPC6, ATP2B1, TRAM2, and PLPP2. By contrast, MDR1, HMGCR, GLUT3, and MCOLN1 seemed largely unaffected in dKO ER membranes (Figures 5B and Figure S5D). Further inhibiting Sec61 with ApraA also had variable effects across substrates (Figure 5B). Very little insertion was seen for PLPP2 and GLUT3 into dKO ER treated with ApraA, with each manipulation having a partial effect. This indicates that for these two substrates, the two Oxa1 family members and Sec61 represent the full complement of insertion options, although, as shown above, the Sec61 route results in an incorrect topology. For TRAM2, ATP2B1, and HMGCR, ApraA had little or no effect unless used with dKO ER, in which case partial inhibition was observed. For CLCN3, TRPC6, and MDR1, ApraA had no obvious effect even in dKO ER (Figure 5B), whereas for MCOLN1, ApraA completely inhibited translocation as expected (Figure S5D).

Taken together, these results paint a complex picture of insertion mechanisms for TMD pairs. First, EMC, and to a lesser extent GEL, contribute to insertion for many TMD pairs, although some substrates like MDR1 and HMGCR are inserted largely unimpaired in their absence. Second, even in cases where substrates are dependent on these Oxa1 family insertases, the requirement is not absolute. Third, some, but not all, substrates can access the Sec61-dependent route of insertion when the Oxa1 family is unavailable, although this route can result in an incorrect single-pass topology. Fourth, most substrates can insert at least partially by a route that is independent of EMC, GEL, and the Sec61 lateral gate. For a substrate like MDR1, insertion is almost entirely unimpaired despite losing access to all three known co-translational insertion routes. Thus, while the data clearly point to a role for the Oxa1 family insertases in the insertion of many TMD pairs, other yet-unidentified mechanisms seem to exist. Consistent with this idea, a recent study showed that yeast Snd3 may be a previously unappreciated insertase.^57^ Although clear homologs of Snd3 outside of fungi remain to be found, similar types of proteins may yet be discovered.

### Sec61-independent TMD-pair insertion by a Sec61-docked ribosome

**Figure 6:**
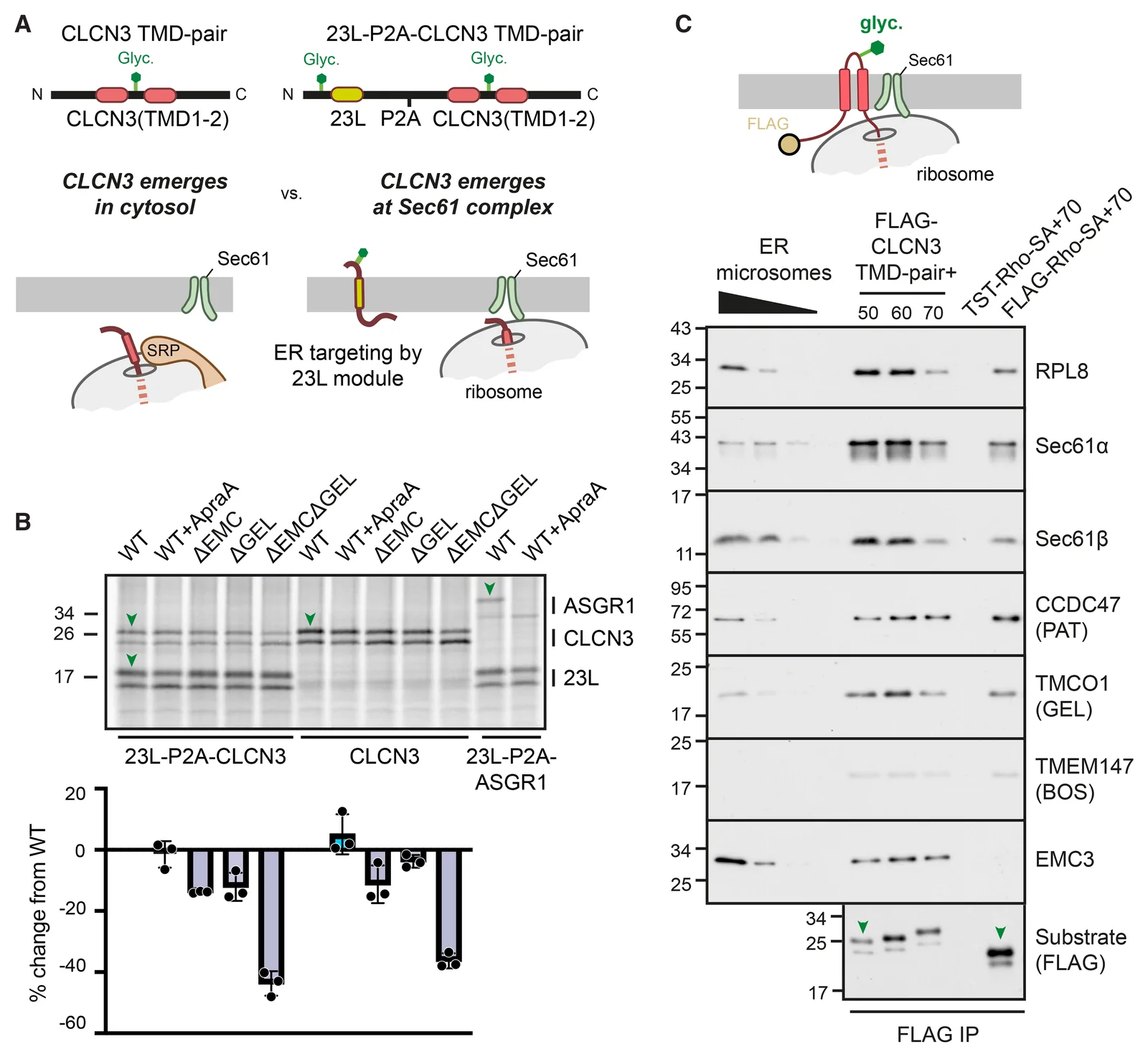
EMC and GEL can insert TMD pairs at a Sec61-docked ribosome (A) Experimental strategy to program the emergence of TMD1 of CLCN3 before (left) or after (right) targeting the Sec61 complex. When preceded with an artificial TMD comprised of 23 leucines (23L), a linker, and a ribosome-skipping P2A peptide, the CLCN3 TMD pair will emerge from the ribosome after it has already been docked at Sec61. By contrast, the native CLCN3 TMD pair emerges in the cytosol, which is then targeted to the ER membrane by SRP. (B) Translocation assays comparing 23L-P2A-CLCN3, CLCN3 TMD pair, or 23L-P2A-ASGR1 using SPCs from the indicated cells, without or with ApraA. Glycosylated products of 23L, CLCN3, and ASGR1 are indicated by green arrowheads. Bottom: quantification of percent change in translocation efficiency in each condition relative to that observed for WT SPCs (mean ± SD; n = 3). (C) Top: cartoon of CLCN3 TMD-pair RNCs with an N-terminal FLAG tag for affinity purification. Bottom: CLCN3 TMD-pair RNCs stalled at the indicated lengths downstream of TMD2 were translated in the presence of WT SPCs. A FLAG-tagged rhodopsin signal anchor (SA) RNC that was stalled 70 amino acids after TMD1 (+70) was included as a positive control for recruitment of the MPT. TST-tagged rhodopsin SA+70 was included as a negative control. Pelleted SPCs were solubilized, and RNCs were purified via the FLAG tag under native conditions. Eluate fractions were blotted for the indicated proteins.

TMD pairs that begin a multipass protein would mediate ER targeting and could have access to EMC before docking at the Sec61 translocation channel.^56^ The fate of TMD pairs that have not inserted by the time of Sec61 docking, or later TMD pairs that emerge from a Sec61-docked ribosome (such as the TMD2-TMD3 pair of all GPCRs), is unclear. To investigate the insertion mechanism of TMD pairs at the ribosome-Sec61 complex, we designed a fusion construct in which targeting and docking at Sec61 are initiated by an N_exo_ TMD, after which the CLCN3 TMD pair is synthesized (Figure 6A). Importantly, the initiating N_exo_ TMD (an artificial 23-leucine TMD) is followed by the ribosome-skipping P2A sequence, allowing the downstream TMD pair to emerge from a ribosome that has already been targeted and docked at Sec61.

Pre-docking at Sec61 in this manner had no impact on insertion of the CLCN3 TMD pair relative to the native CLCN3 TMD pair (Figure 6B). Insertion of the pre-docked construct was completely resistant to ApraA, indicating that even when produced at Sec61, CLCN3 does not need to use Sec61’s lateral gate for insertion. Instead, insertion still relied substantially on Oxa1 family members, showing a clear impairment in dKO ER membranes, with modest effects of losing only EMC or only GEL. As expected, pre-docking the model N_cyt_ substrate ASGR1 in the same manner resulted in its efficient insertion via the Sec61 lateral gate (and hence, was strongly inhibited by ApraA), illustrating that pre-docking does not somehow preclude Sec61 engagement.

Interestingly, whereas native CLCN3 showed no impairment in GEL-KO ER membranes, pre-docked CLCN3 showed a small but detectable impairment. Both showed a small effect of EMC loss. This suggests that CLCN3 has access to both EMC and GEL, consistent with their partial redundancy as insertases, but that the pre-docked situation may rely slightly more on GEL. Consistent with access of CLCN3 to both insertases, we found that affinity purification of membrane-docked RNCs of CLCN3 efficiently recovered both EMC and the multipass translocon (MPT) containing GEL (Figure 6C). By contrast, an RNC intermediate of rhodopsin at a point after insertion of its first TMD recovered the MPT but not EMC. These results indicate that whereas the MPT is recruited to nearly all membrane-bound ribosomes producing multipass proteins,^34^ EMC recruitment may be context and substrate dependent. The full scope of substrates that trigger EMC recruitment to the ribosome remains to be studied.

### An internal TMD pair can be inserted by EMC

Sec61-docked CLCN3 seems to be capable of both recruiting EMC to the ribosome and using it for insertion, despite EMC being restricted to a position ~100 Å away from the ribosome exit tunnel due to steric constraints.^58^ This distance can be reached by the translocating loop of a TMD pair once around 10–15 amino acids downstream of TMD2 have emerged from the ribosome. This raised the possibility that, in addition to inserting TMD(s) at the beginning and end of a multipass protein,^47,48,56^ EMC could mediate insertion of internal TMD pairs at a ribosome-Sec61 complex.

**Figure 7:**
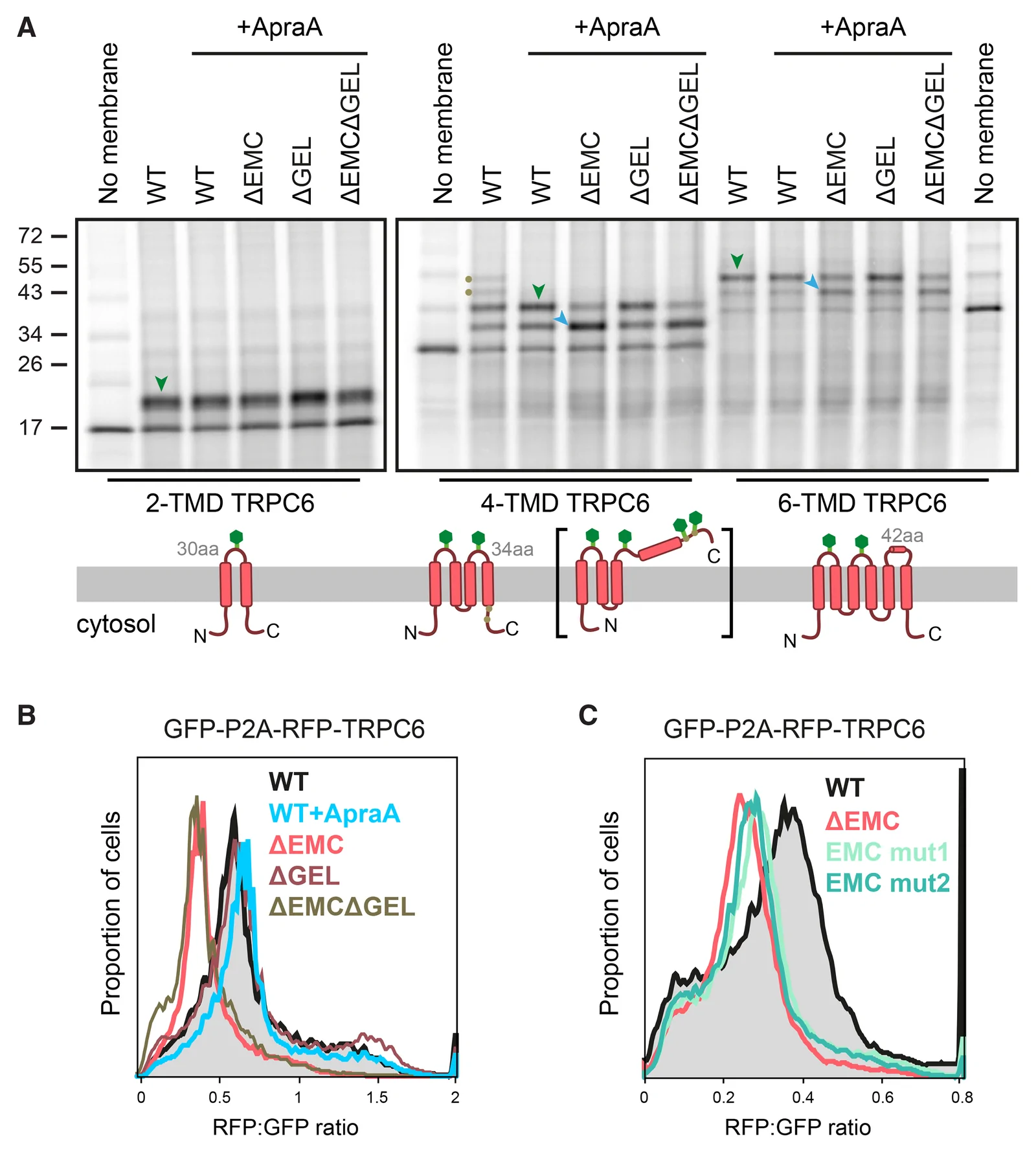
EMC can insert internal TMD pairs (A) Translocation assays of TRPC6 constructs containing the first two, first four, or all six TMDs as indicated below the gels. Each construct was translated in RRL with ^35^S-methionine and the indicated SPCs, with or without ApraA, as indicated. Glycosylated products at each length are indicated with green arrowheads. Yellow dots label hyper-glycosylated forms of the four-TMD construct resulting from translocation of TMD4, as depicted in the diagram in brackets. Blue arrow-heads in the four-TMD and six-TMD constructs indicate an increase in the singly glycosylated species at the expense of the doubly glycosylated product observed in ΔEMC SPCs. (B) Stability of TRPC6 in cells was monitored by flow cytometry using a dual-color TRPC6 fluorescent reporter. WT or indicated KO cell lines were transfected with an inducible construct coding for GFP-P2A-RFP-TRPC6. 16–24 h after transfection, reporter expression was induced with doxycycline for 6 h in the absence or presence of ApraA. The ratio of RFP to GFP as measured by flow cytometry was plotted as a histogram from at least 10,000 cells. (C) The dual-color TRPC6 fluorescent reporter was transfected into WT, ΔEMC, and EMC mutant cells and analyzed as in (B).

To investigate this, we analyzed TRPC6, a multipass protein whose first TMD pair can be inserted in an EMC-independent manner (see Figure 5B), but whose overall biogenesis is dependent on EMC.^59^ Insertion reactions with a construct containing only the first TMD pair showed no impairment of insertion with ApraA or EMC-KO as judged by glycosylation of the translocated loop (Figure 7A, left). Analysis of a construct containing two TMD pairs (i.e., the first four TMDs; Figure 7A, middle) showed that insertion was now substantially dependent on EMC. This was due to impaired insertion of the second TMD pair, which also contains a glycosylation site. Hence, the doubly glycosylated species (green arrow), indicative of both TMD pairs being inserted, was reduced, with a corresponding increase in the singly glycosylated species (blue arrow) representing insertion of only the first TMD pair.

Similar results were seen with the complete construct with all three TMD pairs (Figure 7A, right). These experiments were performed in the presence of ApraA because, in its absence, a pro-portion of the second TMD pair evidently engages Sec61, which inserts TMD3 due to its high hydrophobicity but fails to insert the more hydrophilic TMD4. Translocation of TMD4 and its down-stream domain is evident by the use of two consensus glycosylation sites in this normally cytosolic region (highlighted by yellow dots in lane 8, Figure 7A; see diagram below the gel). This aberrant fate (similar to the aberrant handling of the TMD pairs from PLPP2 and GLUT3 described above) is probably minimized normally due to recruitment of the PAT complex during TRPC6 biogenesis, which prevents Sec61 from opening.^33^

This minor complication notwithstanding, the results indicate that, like an initial TMD pair, an internal TMD pair can use EMC for insertion at a Sec61-docked ribosome. Furthermore, the results underscore the conclusion that insertion of some TMD pairs via Sec61 may be less effective and more prone to error than via Oxa1 family members. To test whether insertion of the second TMD pair of TRPC6 via EMC follows a similar path as other EMC substrates, we analyzed TRPC6 biogenesis in the EMC insertase mutants discussed earlier (see Figure 4A). Stability of fluorescent full-length TRPC6 reporter transfected into cells was unaffected in the presence of ApraA or in GEL-KO cells (Figure 7B). By contrast, TRPC6 biogenesis was impaired in EMC-KO cells. Mutations in EMC3’s cytosolic methionine loop and hydrophilic groove phenocopied EMC-KO cells (Figure 7C), indicating that this insertion path is important for TRPC6 biogenesis.

## Discussion

Our study defines the TMD pair as a major and ubiquitous biosynthetic unit of multipass membrane proteins and establishes that their insertion usually occurs by Sec61-independent mechanisms. TMD-pair insertion as a unit, rather than sequential TMD insertion via the Sec61 lateral gate, departs from the long-standing paradigm for membrane protein insertion.^7,8^ Instead, we find that at least one route is via the Oxa1 family insertases, which in the mammalian ER are GET,^51^ EMC,^39^ and GEL.^33^ Because Oxa1 family insertases are ubiquitous in most major biosynthetic membranes^60–63^ and have existed since the last universal common ancestor,^64^ our findings can likely be extrapolated across the tree of life. The model we propose, based on our findings (see below), resolves a number of otherwise disparate and somewhat contradictory observations and is consistent with a recently proposed unifying model for membrane protein biogenesis.^37^

Based on the structures and earlier mechanistic work on the Oxa1 family insertases,^3,65^ we propose the following working model for TMD-pair insertion. For a TMD pair at the beginning of a protein, the first TMD would emerge from the ribosome, engage the signal recognition particle, target to the ER, and release from SRP with the aid of the SRP receptor and TMEM208.^66,67^ The hydrophobicity of TMD1 (even if only partial) would cause its binding to the membrane surface similar to an amphipathic helix, with insertion being precluded by its flanking hydrophilic domains.^68,69^ In some cases, TMD1 may quickly engage Sec61’s lateral gate, but this is not an obligate step (i.e., completely resistant to ApraA) and is inefficient for some (and perhaps many) non-hydrophobic TMDs such as that from CLCN3. Continued synthesis would allow TMD2 to emerge, which, due to its hydrophobicity, would also bind the membrane surface and possibly interact with TMD1. TMD-TMD interaction prior to insertion seems plausible, given that the two TMDs of nearly all TMD pairs interact with each other in the final structure.^70^

A TMD pair would be more hydrophobic than each TMD alone, especially if their hydrophilic surfaces face each other, as is typical for TMD-TMD interactions.^70–73^ This would therefore favor their membrane partitioning, with the major barrier to insertion being translocation of the hydrophilic loop between the TMDs. This barrier would be lowered by the hydrophilic vestibule of Oxa1 insertases, which locally distorts or thins the membrane,^54,74–76^ to facilitate translocation. This is analogous to the proposed insertion mechanism of tail-anchored or N_exo_ TMDs, where tail translocation is thought to occur via the hydrophilic vestibule of either GET or EMC in eukaryotes^3,48,56,76^ and YidC in bacteria.^74^ Thus, all TMDs flanked by a short translocated domain would insert via a thinned membrane generated in proximity to Oxa1 family insertases. A shared mechanism explains why the length constraint for the translocated tail or loop is roughly 50 amino acids for each situation.

If the translocated loop in a TMD pair is artificially lengthened, insertion either becomes obligately Sec61-dependent (e.g., as seen with TRAM2) or fails completely for low-hydrophobicity TMD1 (e.g., as seen with CLCN3). It seems likely that a long loop between TMD1 and TMD2 is “sensed” by EMC’s inability to accommodate it within EMC’s cytosol-facing vestibule. EMC does not contain a membrane-spanning channel, so there is no stable way for the first TMD to be inserted in an N_cyt_ topology such that a long downstream hydrophilic segment passes back through the membrane shielded from lipids. By contrast, this is accomplished by Sec61 because the long hydrophilic segment passes through its central channel.^10,14^ Short loops are accommodated by EMC because they can be partially housed within EMC’s vestibule until TMD2 emerges and inserts as a pair with TMD1. Thus, the loop-length-dependent choice between EMC and Sec61 may simply be a consequence of their different architectures.

TMD-pair insertion can occur either before or after ribosome docking on Sec61. Insertion prior to docking is clearly possible, given that insertion occurs unimpeded in Sec61-lacking membranes. Insertion at a Sec61-docked ribosome would be the likely route for all internal TMD pairs, which would typically emerge in the context of the MPT containing the GEL complex.^33,34,49^ Importantly, the PAT complex of the MPT prevents Sec61 from opening, redirecting the TMD pair for insertion by another route.^33^ N_cyt_ multipass proteins with only short translocated loops could insert entirely as TMD pairs, with the first one being inserted by EMC and later ones being inserted at the MPT. N_exo_ multipass proteins with only short translocated loops, such as most GPCRs, would also insert their first TMD using EMC,^47,56^ after which the MPT is assembled.^33,49^ The remaining TMDs would then insert as pairs at the MPT, explaining why such GPCRs are refractory to Sec61 inhibitors. In addition to GEL, we find that EMC is also available for the insertion of Sec61-docked substrates, suggesting that EMC can sample the ribosome-Sec61-MPT complex to form a transient supercomplex.

GEL is found in a more limited set of species than EMC.^64^ This is consistent with EMC KOs showing more of an effect on the substrates tested here, although GEL seems to be capable of partially substituting, as evidenced by a greater impairment in dKO cells than in EMC-KO cells. Presumably, EMC and GEL have overlapping but somewhat different substrate preferences, not unlike the overlapping substrate specificities of GET and EMC for tail-anchored proteins.^39^ One might expect GEL to be the major insertase for internal TMDs, given that it is part of the MPT that associates with ribosomes translating multipass membrane proteins.^33,34,49^ EMC seems to be recruited to this complex for co-translational TMD-pair insertion of some substrates, as shown here for TRPC6.

Our *in vitro* findings, together with recent work on the MPT, rationalize why the biogenesis of a large proportion of multipass membrane proteins is refractory to Sec61 inhibitors in cells. This was seen in focused reporter assays on a diverse range of multipass proteins^33,34,49^ and in proteomic analyses of endogenous proteins.^77–79^ The inhibitor-sensitive substrates are primarily those proteins that contain large extracellular domains, either downstream of a signal peptide, between TMDs, or at the C terminus. In each case, translocation of this domain must occur via an aqueous channel, and Sec61 would seem to be the only such conduit. TMDs flanking long translocated domains are generally more hydrophobic than those in TMD pairs, presumably reflecting a requirement for opening the Sec61 channel. Thus, translocation of long domains is strictly dependent on Sec61 and inhibited by lateral gate blockers. By contrast, N_exo_ TMDs with short tails, TMD pairs with short loops, and C-terminal TMDs with short translocated tails are generally Sec61-independent and, to varying degrees, dependent on the Oxa1 family insertases, which lack a membrane-spanning channel and cannot accommodate long translocated loops.

The dispensability of Sec61 for membrane proteins lacking long translocated domains is evident by the lack of a Sec61 homolog in the inner mitochondrial membrane of nearly all species.^80^ Having likely evolved from an alpha-proteobacterium,^81,82^ the ancestral mitochondrion would have had SecY, the bacterial Sec61 homolog. But as the mitochondrion lost most of its protein-coding genes to the nucleus and retained only those for a few membrane proteins, SecY was lost in all but a few species. By contrast, Oxa1 is always retained and is essential for the insertion of mitochondrially encoded membrane proteins, many of which are multipass. This argues that of the two translocation routes, the one favored for membrane protein insertion is the Oxa1 family, with the SecY family being crucial for soluble domain translocation. This matches with the situation in bacteria, where substrates that are solely dependent on YidC, an Oxa1 family member, seem to lack long translocated domains.^62,83^

Sec61 inhibition in EMC/GEL dKO cells revealed that some TMD pairs (e.g., those from MDR1 or HMGCR) can nonetheless insert remarkably effectively, with others still showing detectable insertion. This probably explains why dKO cells are viable in culture, albeit with an appreciable growth deficit, and points to the existence of an alternative insertion pathway. The simplest explanation would be unassisted insertion, whereby the energetic cost of loop translocation is offset by the favorable partitioning of TMDs into the bilayer, an idea proposed long ago.^6^ Such a reaction might be slower and less efficient but works well enough in the highly confined environment of a Sec61-docked ribosome to produce at least some amount of most membrane proteins. Alternatively, other insertases may exist.^57^ Our identification of substrates that insert without EMC, GEL, or Sec61 should facilitate the search for additional insertion mechanisms.

### Limitations of the study

Our study has several limitations. First, protein translocation and topology are primarily analyzed *in vitro*, warranting future work to directly analyze topology in cells. Second, our corroborative cell-based assays rely on efficient degradation of mis-inserted substrates, which is an indirect readout of failed insertion. Third, we extrapolate principles of TMD-pair insertion from a handful of model substrates, which should be extended with proteome-wide studies in the future. Fourth, KO of insertion factors, such as EMC and GEL subunits, could lead to pleiotropic effects that may confound some interpretations. Finally, although TMD pairs interact with EMC and GEL during or after insertion, establishing sufficiency for TMD-pair insertion requires future reconstitution experiments with purified factors.

## Resource Availability

### Lead contact

Further information and requests for resources and reagents should be directed to and will be fulfilled by the lead contact, Ramanujan Hegde (rhegde@mrclmb.ac.uk).

### Materials availability

Plasmids generated in this study will be available upon request.

## Star★Methods

### Key Resources Table

**Table:** 

REAGENT or RESOURCE	SOURCE	IDENTIFIER
Antibodies		
EMC3/TM111 Recombinant Polyclonal Antibody	Invitrogen	711771; RRID: AB_2716909
Calnexin	Enzo	ADI-SPA-865; RRID: AB_11180747
Sec61α	Gilmore lab	Ref: ^84^ RRID: n/a
Sec61β	Hegde lab	Ref: ^85^ RRID: n/a
RPL8	Abcam	ab169538 RRID: AB_2714187
FLAG-HRP	Sigma	A8592 RRID: AB_439702
CCDC47	Bethyl	A305-100A RRID: AB_2631495
TMCO1	Invitrogen	PA5-43350 RRID: AB_2576547
TMEM147	Invitrogen	PA5-95876 RRID: AB_2807678
Peroxidase AffiniPure Goat Anti-Rabbit IgG (H+L)	Jackson ImmunoResearch	111-035-003 RRID: AB_2313567
Chemicals, peptides, and recombinant proteins		
Bismaleimidohexane (BMH)	Thermo	22330
TransIT 293	Mirus	MIR 2704
m7G(5’)ppp(5’)G RNA Cap Structure Analog	New England Biolabs	S1404L
RNasin® Ribonuclease Inhibitor	Promega	N2515
Amino acid kit	Promega	L9961/L996B
SP6 Polymerase	NEB	M0207L
Creatine kinase	Roche	CK-RO, SKU# 10127566001
Creatine phosphate	Roche	CRPHO-RO, SKU# 10621714001
Fetal bovine serum	Gibco	10270106
Rabbit Reticulocyte Lysate Mix	Green Hectares	N/A
EDTA	VWR	20302.260
DMEM, high glucose, GlutaMAX, pyruvate	Gibco	10569-010
FluoroBrite DMEM	Gibco	A18967-01
Hygromycin B	Thermo Fisher	10687-010
SuperSignal West Pico Chemiluminescent substrate	Thermo Fisher	34080
Doxycycline	Sigma	D9891
NEBuilder® HiFi DNA Assembly Master Mix	NEB	E2621L
Sepharose, Strep-Tactin	IBA	IB212010102-1201-010
Concanavalin A (ConA) resin	Sigma	GE17-0440-03
Lipofectamine™ RNAiMAX transfection reagent	Invitrogen	13778075
PonceauS Solution	Sigma	P-7170-1L
Digitonin	Sigma	300410
DAPI	Sigma	D9542
ATP	Roche	ATPDS-RO, SKU#10519979001
UTP	Sigma	U6875
CTP	Sigma	C1506
GTP	Roche	10106399001
Proteinase K	Fisher	BP1700-100
GlutaMAX	Gibco	35050061
RNaseA	Thermo Scientific	EN0531
Sodium pyruvate	Gibco	11360070
Spermidine	Sigma	S0266
EasyTag L-[35S]-Methionine	Perkin - Elmer	NEG709A001MC
ANTI-FLAG M2 Affinity Gel	Millipore	A2220
PNGase F	NEB	P0704S
SYPRO^™^ Ruby	Invitrogen	S12000
Experimental models: Cell lines		
Flp-In 293 T-Rex Cells WT	Thermo Fisher	R78007
Flp-In 293 T-Rex Cells ΔEMC6	Ref.^39^	N/A
Flp-In T-REx 293 cells ΔTMCO1	Ref. ^49^	N/A
Flp-In T-REx 293 ΔEMC6/ΔTMCO1	This study	N/A
Flp-In T-REx 293 EMC3-FLAG	Ref. ^56^	N/A
Flp-In T-REx 293 EMC3 (216C)-FLAG	Ref. ^56^	N/A
Flp-In T-REx 293 EMC3mut1	This study	N/A
Flp-In T-REx 293 EMC3mut2	This study	N/A
Recombinant DNA		
pcDNA5/FRT/TO-GFP-P2A-RFP-ASGR1	Ref. ^47^	N/A
pcDNA5/FRT/TO-MDR1-GFP-P2A-RFP	Ref. ^67^	N/A
pcDNA5/FRT/TO-GLUT3-GFP-P2A-RFP	This study	N/A
pcDNA5/FRT/TO-PLPP2-GFP-P2A-RFP	This study	N/A
pcDNA5/FRT/TO-GFP-P2A-RFP-SQS	Ref. ^47^	N/A
pcDNA5/FRT/TO-CLCN3-GFP-P2A-RFP	This study	N/A
pcDNA5/FRT/TO-GFP-P2A-RFP-TAAR5	Ref. ^67^	N/A
pcDNA5/FRT/TO-GFP-P2A-RFP-TRPC6	Ref. ^67^	N/A
pcDNA5/FRT/TO-GFP-P2A-RFP-β1AR	Ref. ^47^	N/A
pcDNA5/FRT EMC3(Mut1)	This study	N/A
pcDNA5/FRT EMC3(Mut2)	This study	N/A
pcDNA5/FRT EMC3(216C)	Ref. ^56^	N/A
SP64-1xFLAG-CLCN3 TMD1-2-EGFP (LS1126)	This study	N/A
SP64-HA-TRAM2 TMD1-2-EGFP (LS1046)	This study	N/A
SP64-HA-TRPC6 TMD1-2-EGFP (LSG114)	This study	N/A
SP64-HA-MDR1 TMD1-2-EGFP (LSG98)	This study	N/A
SP64-1xFLAG-ATP2B1 TMD1-2-EGFP (LSG185)	This study	N/A
SP64-1xFLAG-HMGCR TMD1-2-EGFP (LSG180)	This study	N/A
SP64-1xFLAG-PLPP2 TMD1-2-EGFP (LSG184)	This study	N/A
SP64-1xFLAG-GLUT3 TMD1-2-EGFP (LSG143)	This study	N/A
SP64-HA-MCOLN1 TMD1-2-EGFP (LSG116)	This study	N/A
SP64-CLCN3-MCOLN1 loop chimeras	This study	N/A
SP64-23L-CLCN3	This study	N/A
SP64-1xFLAG-CLCN3 TMD1-ADR1a-TMD2-EGFP	This study	N/A
SP64-HA-TRPC6-TMDs1-4-cyt.Sec61β	This study	N/A
SP64-HA-TRPC6-TMDs1-6-cyt.Sec61β	This study	N/A
Software and algorithms		
Adobe Illustrator	Adobe	https://www.adobe.com/uk/creativecloud.html
Fiji	https://fiji.sc/	https://fiji.sc/
GraphPad Prism 8	GraphPad	www.graphpad.com
FlowJo	BD	10.10.0

### Experimental Model and Study Participant Details

#### Cell lines

Flp-In™ T-REx™ 293 cells (female) (ThermoFisher R78007) were cultured in DMEM, high glucose, GlutaMAX™ Supplement, pyruvate (Gibco 10569-010) containing 10% fetal bovine serum (Gibco 10270106). ΔEMC6, ΔTMCO1, ΔEMC6ΔTMCO1 dKOs, and EMC3 long-term overexpression cells were created in Flp-In™ T-REx™ 293 cell background as previously described.^33,39,48,49^ All cells were cultured at 37°C in the presence of 5% CO_2_. All cell lines were routinely verified for relevant knockouts.

### Method Details

#### DNA

All plasmids used were cloned by Gibson assembly or site-directed mutagenesis and verified by sequencing. All DNA fragments were ordered from IDT or Twist Bioscience and not further verified. All cDNAs used for in vitro transcription were based on an SP64 plasmid backbone containing an SP6 promoter and lacking the polyA sequence. The following reporters were used, with filler sequences for length match from mEGFP: CLCN3 (NP_001820.2, 88–171) with accompanying variants replacing the second TMD with GFP, lacking cysteine, or containing single cysteine; TRAM2 (NP_036420.1, 14–119) with accompanying variants replacing the second TMD with GFP; TRPC6 (NP_004612.2, 427–521); MDR1 (NP_000918.2, 25–186); ATP2B1 (NP_001673.2, 85–192); HMGCR (NP_001351116.1, 2–90); PLPP2 (NP_003703.1, 2–88); GLUT3 (NP_008862.1, 2–93); MCOLN1 (NP_065394.1, 46–350); SQS, ASGR1, β1AR, and ZnF sequences were from earlier studies.^27,39,47,56^

Plasmids used for flow cytometry experiments were in pcDNA5/FRT/TO backbone, and containing full length proteins noted above with terminal fusions of the GFP-P2A-mCherry module as described before.^38,39^ The P2A sequence used was ATNFSLLKQAGDVEENPGP. WT and mutant EMC3 long-term expression plasmids were cloned into pcDNA5/FRT backbone with C-terminal TEV cleavage sites and 3XFLAG tag, as described before.^56^

#### Generation of stable cell lines

Stable cell lines were generated using the Flp-in system in Flp-In T-REx 293 cells (Thermo Fisher) according to manufacturer’s protocol. For each cell line, one well of 6-well plate (Corning 3516) with cells at approximately 30% confluency (plated the day before) was transfected with 1800 ng of pOG44 (ThermoFisher V600520) and 200 ng of expressing plasmids (encoding EMC3 mutants or CLCN3 stability reporters). After 48–72 h, cells were diluted in media containing 50 μg/ml hygromycin B (ThermoFisher 10687-010) at no more than 25% confluency for selection of stable cells for two weeks. Isogenic clones were pooled for downstream analysis.

#### Knockdown and knockout

Knockdown of EMC3 or Sec61A1 was achieved through siRNA. For EMC3, 10 pmol of siRNA (Silencer Select s31614) was transfected into fluorescent reporter cell lines (in 12-well dish plated the day before) using 3 μL of Lipofectamine RNAiMAX (ThermoFisher 13778150). Cells were harvested and analyzed after 48 hours. For Sec61A1, 10 pmol of siRNA (Silencer Select s26721) was transfected into wild-type cells (in 12-well dish plated the day before) using 3 μL of Lipofectamine RNAiMAX (ThermoFisher 13778150), followed by a second round of knockdown 48 h later using 2 pmoles of siRNA. Cells were analyzed after a total of 72 hours of knockdown. All knockdowns were compared to control knockdowns using Silencer™ Select Negative Control No. 1 siRNA (Invitrogen, 4390843).

Knockout was achieved using standard CRISPR-Cas9-based methods. For generating EMC6 and TMCO1 double knockout cell line, EMC6 knockout cells^39^ were transfected with ribonucleoprotein complexes containing Cas9-GFP V3 (IDT, cat#10008100) and sgRNA (ACTTGTCTGTCCTGTAAACC) using Lipofectamine RNAiMAX. After 48 hours, single GFP-positive cells were sorted into 96-well plates. Successful knockout clones were used for downstream analysis.

#### Flow cytometry

One day before transfecting fluorescent reporters into cells, cells were plated into 12-well plates (Corning, 353225) to have 30%–50% confluency. 500 ng of indicated plasmids were transfected into cells using 3μL of TransIT®-293 Transfection Reagent (Mirus, MIR 2704) according to manufacturer’s protocols. 16–24 hours after adding the transfection mix, doxycycline (Sigma, D9891) was added to induce expression, together with 200 nM of Apratoxin A (ApraA) or equivalent amount of DMSO, where indicated. Expression was induced for 6 hours and cells were harvested with 0.6 mM EDTA pH=7.2 (VWR, 20302.260). Cells were washed with 1xPBS and resuspended in 500 μL of FluoroBrite DMEM (Gibco, A18967-01) containing 10% FBS, 10 units/mL penicillin, 10 μg/mL streptomycin, 1 mM sodium pyruvate (Gibco, 11360070), 1x GlutaMAX (Gibco, 35050061), and 1μg/mL DAPI (Sigma, D9542). Cell clumps were eliminated by passing through a 70 μm cell strainer (Corning, 352350). Cells stably expressing fluorescent reporters were not transfected and analyzed directly on LSRFortessa. Events representing at least 10,000 transfected cells were collected and analyzed on FlowJo 10.10.0. Example gating strategy is shown in Figure S2B.

#### In vitro transcription and translation

In vitro transcription, translation, and protease protection assays were essentially as described before.^86,87^ In brief, transcription reactions contained 40 mM HEPES, pH 7.6; 0.33 mM m7G(5’)ppp(5’)G RNA Cap Structure Analog (NEB, S1404L); 2 mM spermidine (Sigma S0266); 6 mM MgCl_2_; 10 mM reduced glutathione; 0.5 mM UTP (Sigma, U6875); 0.5 mM ATP (Roche, ATPDS-RO); 0.5 mM CTP (Sigma, C1506); 0.1 mM GTP (Roche, 10106399001); 0.4 U/μl SP6 RNA polymerase (NEB M0207L); 0.8 U/μl RNasin ribonuclease Inhibitor (Promega, N2515); 5–10 ng/μL purified DNA containing SP6 promoter. Transcription reaction was performed at 37°C for 1 hour.

The transcription reactions were added directly to translation reactions at 5% of total volume. Translation reactions contained 34% by volume nuclease-treated crude rabbit reticulocyte lysate (Green Hectares), 10%–25% volume of membrane source [semi-permeabilized cells (SPCs) or ER-enriched membranes, prepared as described below]; 1 mM ATP; 1 mM GTP; 2 mM magnesium acetate; 20 mM HEPES, pH=7.4; 1 mM reduced glutathione; 50 mM potassium acetate; 0.3 mM spermidine (Sigma S0266); 12 mM creatine phosphate (Roche, CRPHO-RO); 0.04 mg/mL creatine kinase (Roche, CK-RO); 0.05 mg/mL tRNA (purified from pig liver); 40 μM each of 19 amino acids except for methionine (Promega, L9961), 0.5 μCi/μl EasyTag™ L-[^35^S]-methionine (PerkinElmer, NEG709A001MC). Where indicated, 0.2 mM Zn^2+^ or 2 μM Apratoxin A (or equal amount of DMSO) were added. Translation reaction was performed at 32°C for 30 minutes then put on ice for subsequent manipulations.

In assays of protein topology by glycosylation, the translation reactions were either analyzed directly by SDS-PAGE, or after first isolating membranes by centrifugation. Centrifugation of SPCs or of ER-enriched membranes was at 20,000 x *g* for 2 min at 4°C. In both cases, the supernatant was discarded and the membrane pellet analyzed by SDS-PAGE. For protease protection assays of protein topology, the translation reactions were incubated for 60 min on ice with 0.5 mg/ml proteinase K (PK). Reactions were terminated by addition of PMSF to 10 mM followed by transfer to a tube containing 10-volumes of 1% SDS, 0.1 M Tris, pH 8 preheated to 100°C. After cooling, the samples were processed for either SDS-PAGE, immunoprecipitation, or glycoprotein enrichment via binding to Conconavalin A (ConA) as described below.

#### Preparation of semi-permeabilized cells

Cells cultured in 10 cm dishes or 6-well plates were grown to approximately 70% confluency and harvested by trypsinization. Pelleted cells were incubated in 50 mM HEPES, pH=7.6, 100 mM KOAc, 2.5 mM Mg(OAc)_2_, and 0.01% purified digitonin on ice for 10 min. Cells were pelleted, washed, and resuspended on ice to 4×10^7^ cells/mL in 25 mM HEPES, pH=7.6, 50 mM KOAc, 1.25 mM Mg(OAc)_2_. SPCs were kept on ice and used immediately without freezing.

#### Preparation of ER-enriched membranes

Ten 15 cm dishes of confluent cells were harvested by trypsinization and pelleted cells were resuspended in 5 volumes of ice-cold 20 mM HEPES, pH=7.4; 1.5 mM MgCl_2_; 5 mM KCl; 2 mM DTT; 1x protease inhibitor (Roche, 10106399001). Cells were incubated on ice for 15 minutes and lysed by 35 strokes of Dounce homogenization (DWK Life Sciences 357542). Lysed cells were adjusted to 20 mM HEPES, pH 7.4; 70 mM sucrose; 210 mM mannitol; 0.5 mM EDTA; 2 mM DTT; 1x protease inhibitor. ER membranes were enriched by differential centrifugation at 4°C. First, cell debris and nuclei were eliminated by two 700 x *g* spins, 10 minutes each. Next, membranes were pelleted by 8500 x *g*, and resuspended in 20 mM HEPES, pH 7.4; 70 mM sucrose; 210 mM mannitol; 0.5 mM EDTA; 2 mM DTT; 1x protease inhibitor at OD280=20. Membranes were flash-frozen in liquid nitrogen and stored at -80°C until use in translocation assays.

#### Site-specific chemical crosslinking

20 μL of translation reactions producing ribosome nascent chain complexes of CLCN3 cysteine variants were used for chemical crosslinking. After translation, membranes were pelleted, washed once with 50 mM HEPES, pH=7.6, 100 mM KOAc, 5 mM Mg(OAc)_2_ and resuspended in 25 mM HEPES, pH=7.6, 50 mM KOAc, 2.5 mM Mg(OAc)_2_. Half of the volume was digested with 50 μg/mL RNase A in the presence of 10 mM EDTA on ice for 10 min and analyzed directly. The other half was subjected to BMH (Thermo Scientific 22330) crosslinking at 250 μM final concentration on ice for 10 min. BMH was quenched with 25 mM DTT. The samples were then analyzed after similar RNase treatment. Where indicted, samples were further treated with PNGase (New England Biolabs) according to the manufacturer’s instructions. In some cases, samples were subjected to immunoprecipitation.

#### Immunoprecipitation

For native immunoprecipitation of EMC after crosslinking reactions, membranes were solubilized in 50 mM HEPES, pH=7.4, 200 mM NaCl, 2 mM Mg(OAc)_2_, 1% TX-100 on ice for 10 min. Solubilized membrane was cleared by ultracentrifugation at 100,000 rpm in the TLA 120.1 rotor at 4C for 30 minutes. Cleared membranes were subjected to FLAG immunoprecipitation with ANTI-FLAG® M2 Affinity Gel (Millipore A2220) for 90 minutes at 4C. Beads were washed three times with solubilization buffer and EMC was eluted by boiling or with 0.25 mg/mL 3xFLAG peptide in 50 mM HEPES, pH=7.4, 200 mM NaCl, 2 mM Mg(OAc)_2_, 0.05% TX-100.

Denaturing immunoprecipitation or pulldowns of glycoproteins or products with a twin-strep-tag (TST) were performed on pelleted membranes solubilized in 1% SDS, 100 mM Tris, pH=8, and heated to 100°C for 5 min. The denatured samples were diluted 10 times with 1xPBS supplemented with 250mM NaCl, 0.5% TX-100, 10mM imidazole, then incubated with either 2 μl antiserum plus 5 μL protein A resin, 5 μL ConA resin, or 5 μL StrepTactin resin for 3 hours at 4C. Beads were washed twice with 1xPBS, 250mM NaCl, 0.5% TX-100, 10mM imidazole, and eluted by boiling in SDS-PAGE sample buffer.

#### Affinity purification of RNCs

Purification of RNCs via the nascent chain under non-denaturing conditions was performed exactly as described previously.^33^ In brief, membranes from the IVT reactions were recovered by centrifugation at 4 °C in a TLA-55 rotor (Beckman) for 20 min at 55,000 rpm. The pellet was washed three times with 1× RNC buffer (50 mM HEPES-KOH pH 7.5, 200 mM KOAc, 5 mM Mg(OAc)_2_) then resuspended in one-fourth the volume of the original translation reaction. Resuspended microsomes were diluted eightfold in solubilization buffer (1× RNC buffer supplemented with 1.5% digitonin) and incubated for 10–30 min on ice. Insoluble material was sedimented for 15 min at 20,000 g and 4 °C in a microcentrifuge, and the supernatant transferred to 20 μl of anti-FLAG-M2 affinity resin (Sigma-Aldrich) previously equilibrated in 1× RNC buffer supplemented with 0.25% digitonin (wash buffer). After 2 h with gentle end-over-end rotation at 4 °C, beads were washed three times with wash buffer then transferred to a new tube. The anti-FLAG resin was eluted with 0.25 mg/ml 3× FLAG peptide (Sigma-Aldrich) in wash buffer at 22 °C for 30 min with agitation. Eluates were analysed by SDS–PAGE and immunoblotting, with antibodies indicated in Figure 6C.

#### SDS-PAGE, autoradiography, and Western blotting

Cell lysates or denatured membranes were subjected to Tris-tricine-based SDS-PAGE at 100 V for 60 minutes. Gels were stained, dried and subjected to autoradiography or transferred to 0.2 μm nitrocellulose membranes for blotting. Membranes were blocked by 5% non-fat milk in PBST (0.1% Tween-20) at room temperature for 30 minutes. Blocked membranes were incubated with antibodies (EMC: Invitrogen, 711771, 1:5000; calnexin: Enzo ADI-SPA-865, 1:5000; Sec61α: homemade,^84^ 1:5000; Sec61β: homemade,^85^ 1:5000; RPL8: Abcam, ab169538, 1:10000; FLAG-HRP: Sigma, A8592, 1:5000; CCDC47: Bethyl, A305-100A, 1:10000; TMCO1: Invitrogen, PA5-43350, 1:200; TMEM147: Invitrogen, PA5-95876, 1:2000). With the exception of FLAG-HRP incubated blots, washed blots were incubated with secondary antibodies peroxidase AffiniPure goat anti-rabbit IgG (H+L) (Jackson ImmunoResearch 111-035-003) for 1–2 hours.

#### Loop length analysis

For GPCRs and multi-spanning membrane proteins, translocated loops were identified according to their topology^48^ as follows. The amino acid sequences, positions, and orientations of all TMDs in GPCRs and all TMDs in non-GPCR multi-spanning proteins were extracted from the AFTM database.^88^ From these sets of TMDs, we then pulled out all pairs of TMDs linked by a non-cytosolic (i.e., translocated) loop. The lengths of translocated loops were determined by counting the number of amino acids between the end of the first TMD of the pair and the beginning of the next TMD. All pairs of TMDs separated by a long (>100aa) translocated loop were separated from those with short (<50aa) translocated loops. We then calculated each TMD’s hydrophobicity (ΔG_app_ in kcal/mol) using the ΔG predictor web interface (https://dgpred.cbr.su.se/index.php?p=TMpred)^18^ for all TMD sequences in the long-loop subset and all TMDs in the short-loop subset. Finally, histograms of ΔG_app_ were plotted for the long-loop subset of TMDs and short-loop subset of TMDs for all GPCRs and for all multi-spanning membrane proteins.

### Quantification and Statistical Analysis

All experiments were repeated multiple times. Band intensities of autoradiographs were quantified after background subtraction. Percent translocation was calculated as the percent of total products (glycosylated and unglycosylated) that is glycosylated. When comparing between groups, two-tailed Welch’s t-tests were performed.

## Supplementary Material

Supplemental information can be found online at https://doi.org/10.1016/j.molcel.2026.06.006.

Figs S1-S5

## Data Availability

This study did not analyze any datasets. This paper does not report original code. Any additional information required to reanalyze the data reported in this paper is available from the lead contact upon request.

## Highlights

- The TMD pair is a membrane protein unit of two TMDs and a short non-cytosolic loop
- Almost 90% of the ~18,000 TMDs in human multipass proteins are part of TMD pairs
- Membrane insertion of TMD pairs is independent of the Sec61 translocation channel
- The Oxa1-family insertases EMC and GEL can facilitate TMD-pair insertion

## In brief

Multipass membrane proteins are mostly comprised of transmembrane domain (TMD) pairs, two TMDs linked by a short non-cytosolic loop. Smalinskaitė et al. show that TMD pairs are inserted as a unit by the Oxa1-family insertases EMC and GEL, not sequentially through the Sec61 protein translocation channel as was previously thought.
