# Supplementary material for: Pairwise transmembrane domain insertion during multipass protein biogenesis
Source: Mol Cell. Author manuscript; Available in PMC 2026 Sep 8. (PMC7619463; doi:10.1016/j.molcel.2026.06.006)
Supplement: Supplementary file 1 [file EMS218175-supplement-Figs_S1_S5.pdf]

Molecular Cell, Volume *86*

Supplemental information

Pairwise transmembrane domain insertion during multipass protein biogenesis

Luka Smalinskaitė, Haoxi Wu, and Ramanujan S. Hegde

A

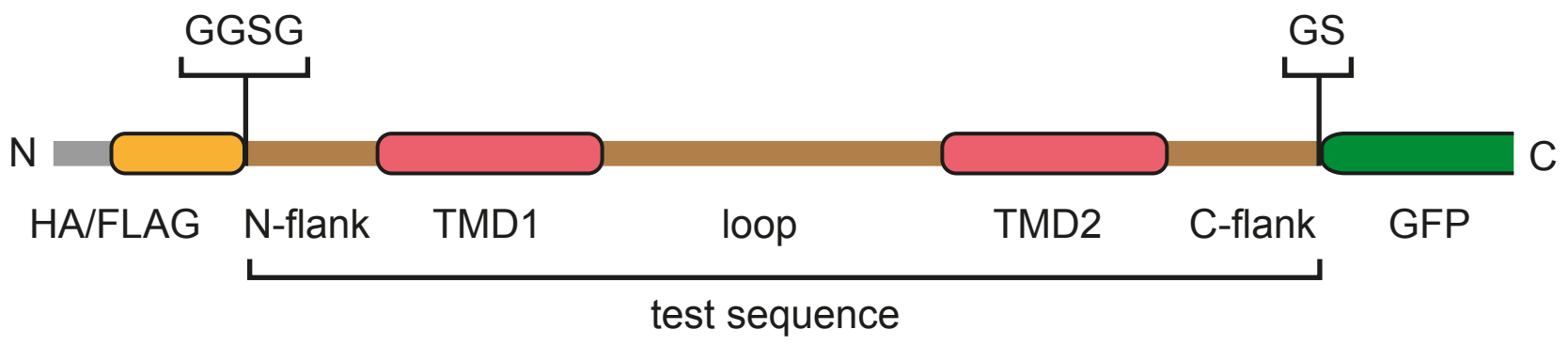

B

name	test sequence[1]	minimum & actual ΔGapp1 (kcal/mol)[2]	minimum & actual ΔGapp2 (kcal/mol)	trans -located length (aa)[3]
CLCN3	FHTIDWVREKCKDRERHRRINSKKKESAWEMTKSLYDAWSGWLVVTLTGLASGALAGLIDI<br>AADWMTDLKEGICLSALWYNHEQCCWGS**NET**TFEERDKCPQWKTWAELIIGQAEGPGSY<br>IMNYIMYIFWALSFAFLAVSLVKVFAPYACGSGIPEIKTILSGFIIRGYLGK	+0.30<br>+4.67	-1.62<br>-1.21	45
TRAM2	FRRRTKSYPLFSQEFVIHNHADIGFCLVLCVLIGLMFEVTAKTAFLFILPQY**NIS**VPTADSETV<br>HYHYGPKDLVTILFYIFITIMLHAVVQEYILDKISKRLHLSKVKHSKFNESGQ	-1.80<br>+0.08	-0.91<br>-0.32	28
TRPC6	APCSKMGKIMRGPFMKFVAHAASFTIFLGLLVMNAADRFEGTKLLP**NET**STDNAKQLFRMK<br>TSCFSWMEMLIISWVIGMIWAECKEIWTQGPKEY	+0.88<br>+1.09	+0.13<br>+1.91	30
MDR1	KDKKEKKPTVSVFSMFRYSNWLDKLYMVVGTLAAIIHGAGLPLMMLVFGEMTDIFANAGNL<br>EDLMS**NITNRS**DI**NDT**GFFMNLEEDMTRYAYYYSGIGAGVLVAAYIQVSFWCLAAGRQIHKI<br>RKQFFHAIMRQEIGWFDVHDVGELNTRLTDDVSKINEGI	+0.05<br>+0.48	-0.69<br>-0.05	41
ATP2B1	FGKNFIPPKKPKTFLQLVWEALQDVTLIILEIAAIVSLGLSFYQPPEGDNALCG**NDT**EVSVGE<br>EEGEGETGWIEGAAILLSVVCVVLVTAFNDWSKEKQFRGLQSRIEQEQ	+0.05<br>+1.51	-0.23<br>+0.29	25
HMGCR	LSRLFRMHGLFVASHPWEVIVGTVTLTICMMSMNMFTGNNKICGWNYECPK**NDT**FDIFEE<br>DVLSSDIIILTITRCIAILYIYFQFQNLRQLGSKY	+1.86<br>+3.81	+0.14<br>+1.54	23
PLPP2	QRRWVFVLLDVLCLLVASLPFAILTLVNAPYKRGFYCGDDA**NDT**SIRYPYRPDTITHGLMAG<br>VTITATVILVSAGEAYLVYTDRLYSRSDF	-2.46<br>-2.46	+0.47<br>+2.64	22
GLUT3	GTQKVTPALIFAITVATIGSFQFGYNTGVINAPEKIMKEFI**NKT**LTDKGNAPPSEVLLTSLWSL<br>SVAIFSVGGMIGSFSVGLFVNRFGRRNS	+1.02<br>+5.14	-0.24<br>+0.81	25
MCOLN1	KYFFMSPCDKFRAKGRKPCKLMLQVVKILVVTVQLILFGLSNQLAVTFREENTIAFRHLFLL<br>GYSDGADDTFAAYTREQLYQAIFHAVDQYLALPDVSLGRYAYVRGGGDPWT**NGS**GLALC<br>QRYYHRGHVDPA**NDT**FDIDPMVVTDCIQVDPPERPPPPPSDDLTLLESSSSYK**NLT**LKFHK<br>LV**NVT**IHFRLKTINLQSLINNEIPDCYTFSVLITFDNKAHSGRIPISLETQAHIQECKHPSVFQH<br>GDNSFRLLFDVVVMLTCSLSFLLCARSLLRGFLLQNEFVGFMWRQRGRVISLWERLEF	+0.87<br>+1.14	-2.35<br>-1.83	210
SQS	MDYKDHDGDYKDHDIDYKDDDDKAGSPGPTPSGTNVGSSGRSPSKAVAARAAGSTVRQ<br>RKNASCGTRSAGRTTSAGTGGMWRFYHSTGGSSRSHYSPIYLSFVMLLAALSWQYLTTL<br>SQVTEDMNGTEGPNFYVPFS**NKT**VD	-0.24<br>+0.56	n/a	25
ASGR1	MTKEYQDLQHLDNEESDHHQLRKGPPPPQPLLQRLCSGPRLLLLSLGLSLLLLVVVCVIGS<br>QNSQLQEELRGLRETFS**NFT**ASTEAQVKGLSTQGGNVGRKMKSLESQLEKQQKDLSEDH<br>SSLLLHVKQFVSDLRSLSCQMAALQG**NGS**ERTCCPVNWVEHERSCYWFSRSGKAWADA<br>DNYCRLEDAHLVVVTSWEEQKFVQHHIGPVNTWMGLHDQNGPWKWVDGTDYETGFKN<br>WRPEQPDDWYGHGLGGGEDCAHFTDDGRWNDDVCQRPYRWVCETELDKASQEPPLL	-2.63<br>-2.11	n/a	232
β1AR	MAYPYDVPDYATA**NGT**GSRQVSAELLSQQWEAGMSLLMALVVLLIVAGNVLVIAAIGSTQR<br>LQTLTNLFITSLACADLVVGLLVVPFGATLVVRGTWLWGSFLCELWTSLDVLCVTASIETLCV<br>IAIDRYLAITSPFRYQSLMTRARAKVIICTVWAISALVSFLPIMMHWWRDEDPQ...	-1.96<br>-1.96	n/a	33

[1]The lowest ΔG_{app} sequences in the regions of the TMDs are underlined. However, the actual TMD boundaries in the membrane are typically different as deduced from the experimental or predicted structures. These structure-based TMDs are in red, and the ΔG_{app} values of these are cited in Fig. 1A. Glycosylation sites are shown in bold type.

[2]Minimum ΔG_{app} is the lowest value obtained for each TMD in a full protein scan using a window of 17 to 30 aa. The actual ΔG_{app} is that for the structure-predicted TMDs shown in red.

[3]Translocated domain length used the red TMD boundaries as determined by the experimental or predicted structure.

Figure S1. Details of key constructs used in this study. Related to Figure 1.

(A) Diagram showing the general organization of the TMD-pair constructs. All constructs begin with an N-terminal epitope tag and a four amino acid linker (GGSG) and end with a two amino acid linker (GS) followed by eGFP.

(B) Annotated sequences of the constructs analyzed in Fig. 1A-C. Explanatory footnotes are shown below the table.

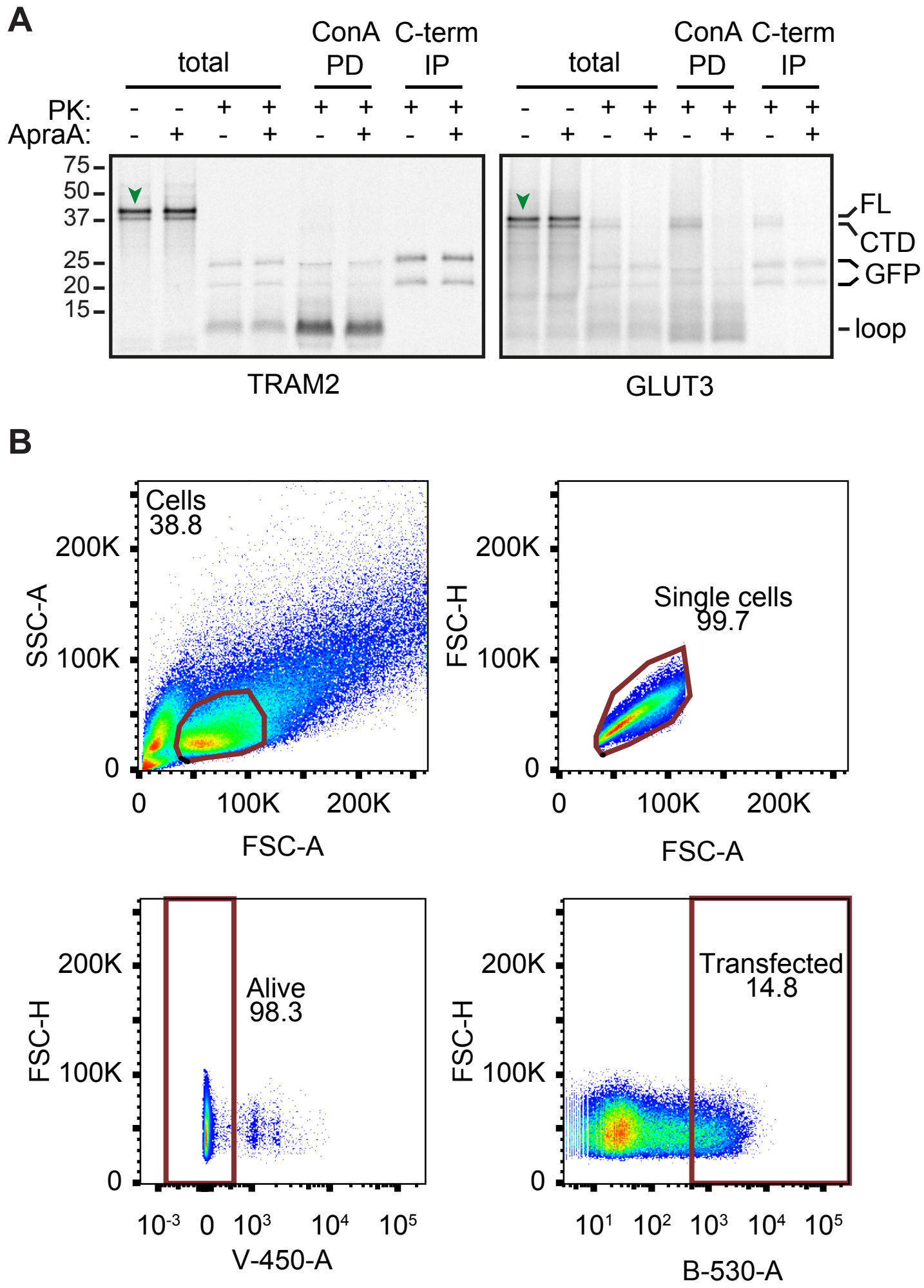

Figure S2. TMD-pair insertion is refractory to Sec61 inhibition. Related to Figure 1.

(A) TRAM2 and GLUT2 TMD-pairs were translated in RRL containing ^{35}S-methionine and ER-enriched membranes in the absence or presence of ApraA. Membranes were pelleted, washed, resuspended, and aliquoted for treatment without or with proteinase K (PK). An aliquot was analyzed directly (total), and the rest was subjected to Concanavalin A pull-down (ConA PD) or GFP immunoprecipitation (C-term IP). Full length product (FL), the protease-protected C-terminal domain (CTD), and the protease-protected TMD-pair fragment (loop) are indicated. The GFP variant used in these constructs is resistant to PK digestion, resulting in two prominent fragments at ~20 and ~25 kD, as indicated. The loop fragment of GLUT3 is somewhat heterogeneous in size and migrates as a smear relative to the more homogeneous fragment from TRAM2 or PLPP2 (see Fig. 1D).

(B) Gating strategy for flow cytometry experiments in this study. Cells, single cells, alive cells, and transfected cells were gated according to FSC area (FSC-A), SSC area (SSC-A), FSC height (FSC-H), V-450 area (V-450-A), and B530 area (B530-A).

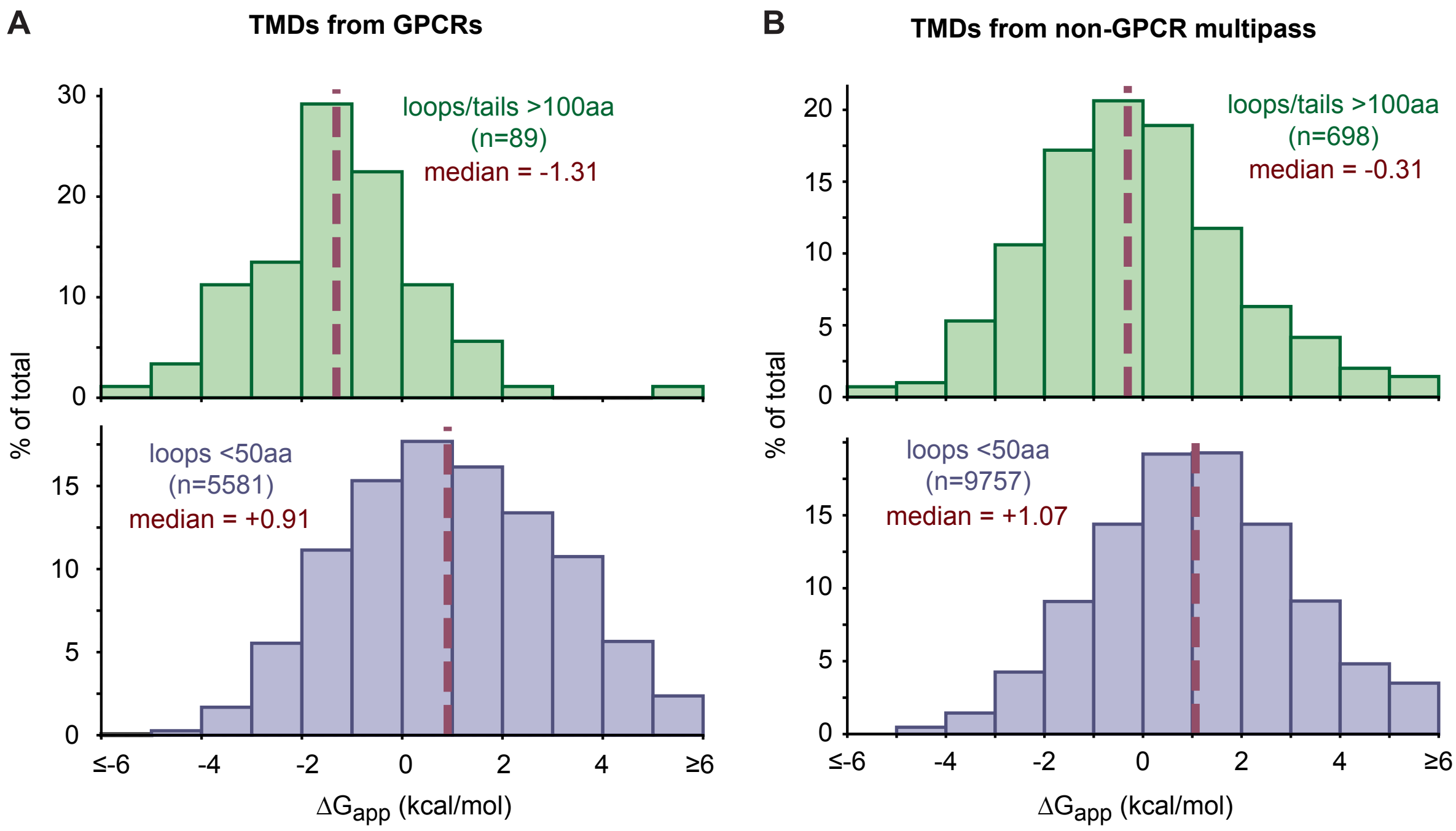

Figure S3. Analysis of TMD hydrophobicity by flanking loop length. Related to Figure 2.

(A) TMDs from GPCRs, whose topology is known with certainty, were grouped according to the length of their adjacent translocated loops or tails. Histograms of the hydrophobicity (calculated by ΔG_{app}, the free energy for membrane insertion in kcal/mol) of TMDs adjacent to loops or tails longer than 100 amino acids or shorter than 50 amino acids were plotted in green and purple, respectively.

(B) TMDs from non-GPCR multipass proteins were analyzed and plotted as in panel A.

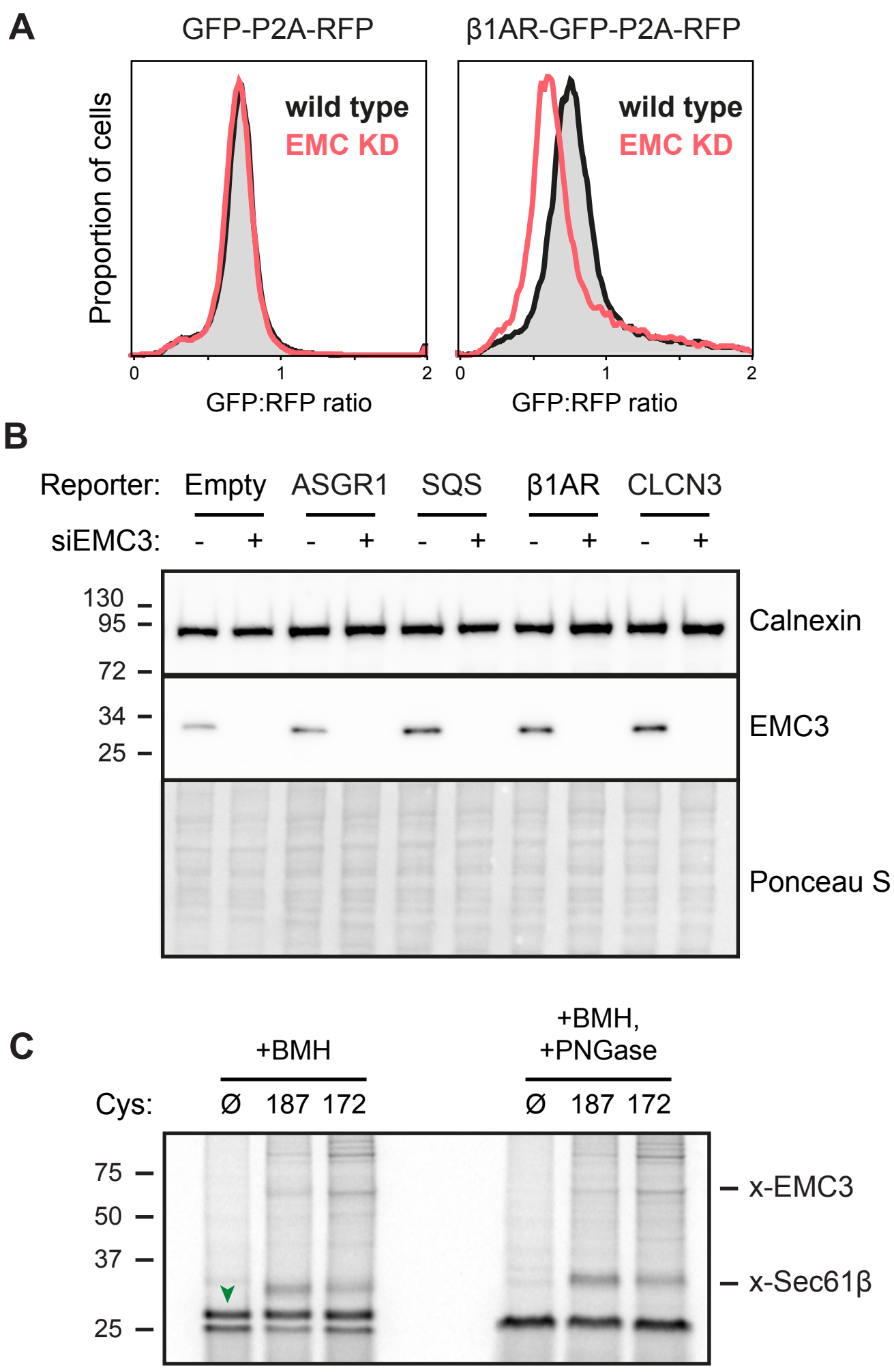

Figure S4. Analysis of EMC knockdown cells and crosslinking. Related to Figure 4.

(A) Stability of dual color fluorescent reporters were analyzed in control and EMC knockdown cells using flow cytometry as in Fig. 4D.

(B) Knockdown efficiency of EMC3 in various reporter cell lines was verified by blotting. Calnexin blot and Ponceau S staining for total protein serve as loading controls.

(C) The CLCN3 TMD-pair was subjected to BMH crosslinking as in Fig. 4F and the samples were analyzed directly or first treated with PNGase to digest all N-linked glycans. Note that although the glycosylated substrate (green arrowhead) shifts to a faster migrating product with PNGase, the crosslinks to Sec61 and EMC3 do not shift, indicating that they are crosslinked to the non-glycosylated (and non-translocated) population of substrate.

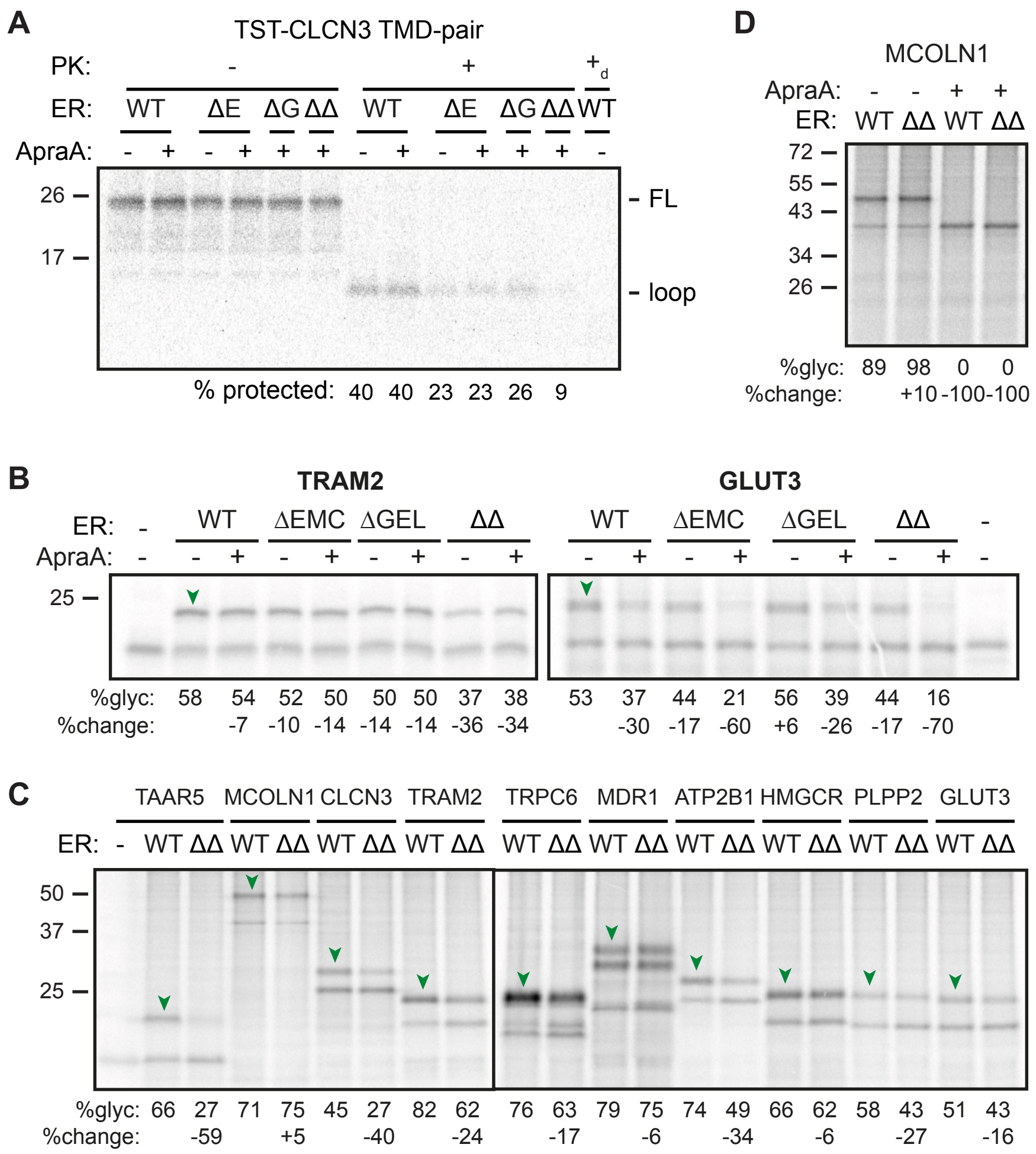

Figure S5. EMC and GEL redundantly facilitate TMD-pair insertion. Related to Figure 5.

(A) Protease-protection assay of a TST-tagged CLCN3 TMD-pair construct in which the TST tag replaces an equivalent length of polypeptide in the loop between TMD1 and TMD2. SPCs isolated from the indicated cell lines were tested without or with ApraA as indicated. +d denotes addition of detergent during PK digestion. All samples were subjected to pulldown via the TST prior to analysis. The protease-protected loop is indicated. Note that this construct does not contain a glycosylation site in the translocated loop.

(B) The TMD-pairs from TRAM2 and GLUT3 were translated in the absence or presence of WT, ΔEMC, ΔGEL, or EMC/GEL double knockout (ΔΔ) SPCs as the source of ER membranes. Samples were analyzed directly (-ER) or after pelleting of membranes. Glycosylated populations are indicated by green arrowheads. Percent glycosylation and percent changes were quantified and indicated below.

(C) The indicated TMD-pairs were translated in the absence (-ER) or presence of WT or EMC/GEL double knockout (ΔΔ) SPCs. Samples were analyzed directly (-ER) or after pelleting of membranes. Glycosylated populations are indicated by green arrowheads. Percent glycosylation and percent changes were quantified and indicated below.

(D) The MCOLN1 construct (see Fig. 1A) was analyzed as in panel A, in the presence or absence of ApraA.
